# The noncoding RNAs SNORD50A and SNORD50B-mediated TRIM21-GMPS interaction promotes the growth of p53 wild-type breast cancers by degrading p53

**DOI:** 10.1038/s41418-021-00762-7

**Published:** 2021-03-19

**Authors:** Xi Su, Chao Feng, Simeng Wang, Liang Shi, Qingqing Gu, Haihong Zhang, Xinhui Lan, Yuelei Zhao, Wei Qiang, Meiju Ji, Peng Hou

**Affiliations:** 1 Key Laboratory for Tumor Precision Medicine of Shaanxi Province and Department of Endocrinology, The First Affiliated Hospital of Xi’an Jiaotong University, Xi’an, 710061 PR China; 2 Center for Translational Medicine, The First Affiliated Hospital of Xi’an Jiaotong University, Xi’an, 710061 PR China

**Keywords:** Cancer genetics, Tumour biomarkers

## Abstract

Small nucleolar RNA SNORD50A and SNORD50B (SNORD50A/B) has been reported to be recurrently deleted and function as a putative tumor suppressor in different types of cancer by binding to and suppressing the activity of the KRAS oncoproteins. Its deletion correlates with poorer patient survival. However, in this study, we surprisingly found that SNORD50A/B loss predicted a better survival in breast cancer patients carrying wild-type p53. Functional studies showed that SNORD50A/B deletion strongly inhibited the proliferation, migration, invasion and tumorigenic potential, and induced cell cycle arrest and apoptosis in p53 wild-type breast cancer cells, while exerted the opposite effects in p53 mutated breast cancer cells. This was also supported by ectopically expressing SNORD50A/B in both p53 wild-type and mutated breast cancer cells. Mechanistically, SNORD50A/B clearly enhances the interaction between E3 ubiquitin ligase TRIM21 and its substrate GMPS by forming a complex among them, thereby promoting GMPS ubiquitination and its subsequent cytoplasmic sequestration. SNORD50A/B deletion in p53 wild-type breast cancer cells will release GMPS and induce the translocation of GMPS into the nucleus, where GMPS can recruit USP7 and form a complex with p53, thereby decreasing p53 ubiquitination, stabilizing p53 proteins, and inhibiting malignant phenotypes of cancer cells. Altogether, the present study first reports that SNORD50A/B plays an oncogenic role in p53 wild-type breast cancers by mediating TRIM21-GMPS interaction.

## Introduction

Breast cancer is the most common malignancy among women worldwide, and about one in eight to ten women is likely to get breast cancer in their lifetime [1, 2]. Benefiting from early detection and efficient systemic therapies [3–5], deaths from breast cancer are decreasing in North America and west European countries [6]. However, breast cancer is still one of the most common causes of cancer-related death in developing countries [1, 7]. Thus, a better understanding of the mechanisms underlying the pathogenesis of breast cancer will lead to more precise prognostic prediction and more effective therapies for this disease.

Coordination of transcription is one of the major responses to various stimulations from carcinogenic factors, which is programmed by p53 that ultimately suppresses tumor growth [8, 9]. Loss of p53 function, mainly by mutations in p53, has been revealed to be a common feature in the majority of human cancers, including breast cancer [10, 11]. Mutation rate of p53 is about 12-28% among ER^+^HER2^-^/luminal breast cancer, while is nearly 80% in the Triple-Negative breast cancers (TNBC), which is the most aggressive subtype of breast cancer [12]. In addition to genetic inactivation, p53 can be functionally inactivated by post-transcriptional modifications such as protein ubiquitination, which is involved in the regulation of protein stability [13–15]. In recent years, different approaches have been developed to inactivate or reconstruct mutant p53, or activate wild-type p53 as potential cancer therapeutic strategies [16, 17].

Small nucleolar RNAs (snoRNAs), a group of conserved noncoding RNAs, are mainly divided into two classes: The H/ACA and the C/D box [18]. Usually, they function as ribonucleoprotein guiding to modify small RNAs such as rRNAs and tRNAs [19, 20]. However, later researches indicate that snoRNAs may play broader roles in human diseases, including malignancies [21, 22]. SNORD50A/B is a pair of snoRNAs with a length of about 70 bases. It is co-located on chromosome 6q14.3 and encodes two C/D box–containing snoRNAs that specify sites for 2’-O-ribose methylation on target RNAs, such as 28S rRNA [23]. Recent studies revealed that *SNORD50A*-*SNORD50B* snoRNA locus was frequently deleted in the different types of cancer, and its loss was associated with poor patient survival. Further studies showed that SNORD50A/B could directly bind to and inhibit the activity of KRAS oncoproteins [24, 25], indicating that it may exert tumor suppressor function in tumorigenesis and tumor progression by suppressing the activity of the KRAS/RAF/MEK/ERK pathway.

TRIM21, an E3 ubiquitin ligase, belongs to the tripartite motif-containing (TRIM) family [26] and plays a key role in the regulation of antibodies mediating intracellular immunity [27, 28]. Further studies have revealed the complex function of TRIM family in human cancers [29, 30]. Guanosine 5′-monophosphate synthase (GMPS) mediates the final step of de novo synthesis of guanine nucleotides, converting xanthosine 50-monophosphate into GMP [31]. Recently, there are studies demonstrating that TRIM21 promotes the progression of various types of cancer by destabilizing p53 proteins via GMPS [32, 33].

In this study, we validate tumor suppressor role of SNORD50A/B in p53 mutated (p53mt) breast cancers; however, we surprisingly find that it plays the complete opposite roles in p53 wild-type (p53wt) breast cancers, and demonstrate that SNORD50A/B induces p53 ubiquitination and degradation by mediating the interaction between TRIM21 and GMPS, thereby promoting the growth of p53wt breast cancers.

## Materials and methods

### Copy number variation and survival curve analysis

Copy number and related clinical data for each sample were downloaded from the TCGA Data Portal on 12 March 2017. Somatic copy number alterations and survival analysis were performed as previously described [25].

### Cell culture

Human breast cancer cell lines MDA-MB-231, T-47D, HCC1937, MCF-7, DU4475, ZR75-1, and human embryonic kidney cell line 293T were obtained and authenticated from the American Type Culture Collection and Shanghai Bioleaf Biotech Co., Ltd. MDA-MB-231, HCC1937, and MCF-7 cells were routinely cultured at 37 °C in RPMI-1640 medium with 10% fetal bovine serum (FBS). DU4475 and 293T cells were cultured at 37 °C in DMEM media medium with 10% FBS. All cell lines were regularly checked to be free of mycoplasma.

### Transfection of antisense oligonucleotides (ASOs), short interfering RNAs (siRNAs), and lentiviruses

ASOs targeting SNORD50A and SNORD50B and control ASO were obtained from RiboBio Co., Ltd. (Guangzhou, China). The siRNAs targeting GMPS (si-GMPS-1 and si-GMPS-2) and control siRNA (si-NC) were also obtained from RiboBio Co., Ltd (Guangzhou, China). The siRNAs targeting USP7 (si-USP7-1 and si-USP7-2) and control siRNA (si-NC) were obtained from Gene Pharma (Shanghai, China). Cells were transfected at 40% confluence using X-tremeGENE siRNA Transfection Reagent (Catalog#: 04476093001, Roche Diagnostics GmbH, Mannheim, Germany) with a final ASO concentration of 60 nM or a final siRNA concentration of 40 nM. The sequences of ASOs and siRNAs were presented in Supplementary Table S1, and were then used to transfect MDA-MB-231, HCC1937, MCF-7, and DU4475 cells. The lentivirus-based CRISPR-Cas9 technique was performed to knock out SNORD50A/B in HCC1937, MDA-MB-231, MCF-7, DU4475, and 293T cells. The detailed protocol and target sequences specific to SNORD50A/B were described as previously [25].

### RNA extraction and quantitative RT-PCR (qRT-PCR)

RNA extraction, cDNA synthesis, and qRT-PCR were carried out as previously described [34]. The mRNA expression of the indicated genes was normalized to 18S rRNA, and the expression of SNORD50A/B was normalized to *U6*. Relative RNA expression was calculated according to 2^−ΔΔCt^ method. Each sample was run in triplicate. The primer sequences were presented in Supplementary Table S2.

### Construction and transfection of expression plasmids

The expression plasmids for TRIM21 and GMPS (pcDNA3.1-TRIM21, pcDNA3.1-HIS- GMPS, pcDNA3.1-Ub) were constructed as described previously [35]. The expression plasmid of SNORD50A/B (pSPARTA-50AB) was kindly provided by Prof. Paul A Khavari (Stanford University School of Medicine, CA, USA). The expression plasmid of p53 (GV141-p53-FLAG) was obtained from Shanghai GeneChem Co., LTD. MDA-MB-231, HCC1937, MCF-7, DU4475, and 293T cells were transfected with the indicated plasmids at 70% confluence using X-tremeGENE HP DNA Transfection Reagent (Catalog#: 06366244001, Roche Diagnostics GmbH, Mannheim, Germany).

### Cell proliferation, colony formation, cell cycle, cell apoptosis, migration, and invasion assays

The detailed protocols were similarly described as previously [36].

### Animal studies

Four- to five-week-old female athymic nude mice were purchased from SLAC Laboratory Animal Co., Ltd. (Shanghai, China) and housed in a specific pathogen-free (SPF) environment. These mice were then randomly divided into four groups according to its number with no blinding (*n* = 5/group). Next, MDA-MB-231 (4 × 10^6^) and MCF-7 (6 × 10^6^) cells stably knocking out SNORD50A/B or control cells were mixed with Matrigel (Corning, NY, USA) and injected subcutaneously into right armpit region of nude mice, respectively, to establish tumor xenografts. Tumor size was then measured every other day from 5 days after injection, and tumor volumes were calculated by the formula: Tumor volume = length × width^2^ × 0.5. The mice were sacrificed at 14 days after injection, and tumors were then weighted and harvested for further exams. The above animal experiments were approved by the Institutional Review Board of Xi’an Jiaotong University Health Science Center.

### Immunohistochemistry (IHC)

IHC assay was performed to evaluate the expression of Ki-67 and p53 in xenograft tumor sections as described previously [34]. The number of positive cells was calculated in five microscopic fields from each group.

### Western blot analysis

The indicated cells were harvested and lysed in the ice-cold RIPA containing protease inhibitors and were then subjected to western blot analysis as described previously [34]. Antibodies used in this study were presented in Supplementary Table S3.

### Cycloheximide (CHX) chase assay

MDA-MB-231 and MCF-7 cells were incubated with 200 µg/mL CHX (MP Biomedicals, CA, USA) for the indicated time after transfecting with ASOs for 48 h to inhibit de novo protein synthesis. Cells were then harvested and lysed for western blot analysis.

### Immunoprecipitation (IP)

The concentration of proteins was adjusted to equal incorporation before immunoprecipitation assay. Next, the lysates were incubated with indicated antibodies or IgG at 4 °C for 4–5 h, followed by incubation with protein A/G-agarose beads (Catalog#: sc-2003, Santa Cruz, CA, USA) at 4 °C overnight. Immunoprecipitates were then washed and further analyzed by western blot analysis. All IP antibodies and IB antibodies used in each immunoprecipitation assay were different resources, and the detailed information of antibodies was presented in Supplementary Table S3.

### Immunofluorescence (IF)

The indicated cells were seeded onto coverslips in a 6-well plate and cultured till 40% confluence. Cells were then fixed with 4.0% paraformaldehyde for 15 min, and blocked with 5% goat serum for 30 min, followed by incubating cells at 4 °C with primary antibodies overnight. Next, the coverslips were incubated with Alexa Fluor 488 or 555-conjugated goat anti-rabbit and goat anti-mouse secondary antibody (Invitrogen, Thermo Fisher Scientific Co., Ltd.) for 1.5 h. Cells were then dyed with Hoechst33342, and kept in glycerol. The images were obtained with a laser scanning confocal microscope (Leica, Wetzlar, Germany), and color emergence was performed using ImageJ image software (ImageJ version 1.44p, NIH, MD).

### Protein expression, refolding, and purification

The cDNAs for human GMPS and TRIM21 were cloned and inserted into the pET-28a (+) plasmid, respectively. The resulting pET-28a(+)-HIS-TRIM21 and pET-28a(+)-HIS-GMPS plasmids were transformed into the *Escherichia coli* BL21(DE3) star bacteria. Ensuing expression of recombinant TRIM21 and GMPS resulted in insoluble inclusion bodies. Bacterial culture was harvested by centrifugation and then lysed by sonication in a buffer containing 50 mM Tris pH 8.0, 200 mM NaCl, 2 mM EDTA, 0.5% Triton X-100, 0.1 mM PMSF, and 1 mM dithiothreitol (DTT). Inclusion bodies were washed several times with a buffer containing 50 mM Tris pH 8.0, 200 mM NaCl, 2 mM DTT, and collected by centrifugation. Next, inclusion bodies were further dissolved in 8 M urea. The solubilized supernatants were subjected to SDS-PAGE, followed by Coomassie Blue staining. Dissolved proteins were then purified with His-tag Purification Resin (Beyotime Biotechnology, Jiangsu, China) and refolded seven times by diluting in 20 mM Tris pH 8.0, 150 mM NaCl (Tris buffer). The solution was dialyzed with Tris buffer at 4 °C for 4–5 times followed by centrifuging at 12,000 rpm for 20 min. The supernatants were concentrated with an Amicon Ultra (Cat: UFC903008, Millipore, MA, USA) and analyzed with a reverse-phase High-Performance Liquid Chromatography (HPLC) on a Waters XBridge C18 column (4.6 × 150 mm, 3.5 μm).

### Fluorescence polarization (FP) assay

FAM-labeled SNORD50A/B and scrambled RNA were obtained from Gene Pharma (Shanghai, China). All fluorescence polarization assays were done using black, low-protein-binding 384-well plates in a total volume of 50 μL per well. Each well contains 5 μM SNORD50A or SNORD50B and gradient diluted proteins (TRIM21 alone, GMPS alone, and TRIM21 + GMPS) with a total concentration from 24 to 0.025 μM in Tris buffer. After a gentle mixing and incubation for 3 h, FP readings were taken at 470 nm (excitation) and 530 nm (emission) wavelengths on a Tecan Infinite M2000 fluorescence plate reader.

### Circular dichroism (CD) spectroscopy assays

The solvent of the protein solution was changed from Tris buffer to phosphate buffer by repeatedly centrifuged in an Amicon Ultra. Next, the proteins were diluted at a loading concentration of 5 μM and grouped accordingly. CD assay was then similarly performed as described previously [37].

### Statistical analysis

Student’s *t*-test, two-way ANOVA with Bonferroni post-test, and one-way ANOVA with Dunnett’s post-test were used for comparing the data. SPSS statistical package (16.0, Chicago, IL) was used to calculate the statistically significance. Experiments were repeated triplicated. The data were expressed as mean ± standard deviation (SD). *P* < 0.05 was considered statistically significant.

## Results

### p53 status is a determinant for the effect of SNORD50A/B deletion on the survival of breast cancer patients

**Fig. 1:**
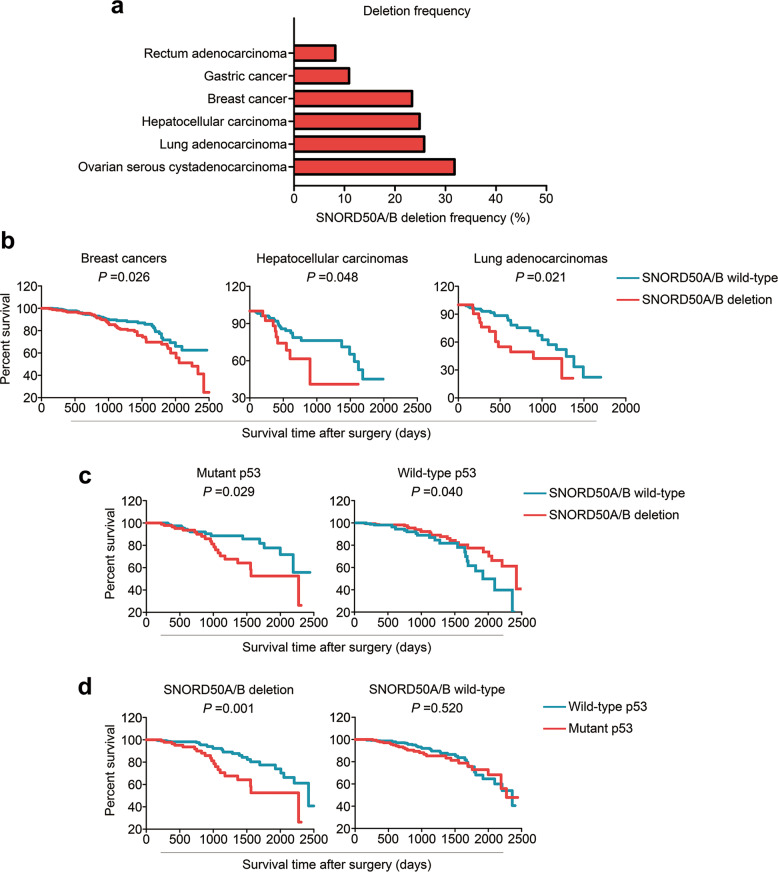
The association of SNORD50A/B deletion with patient survival in breast cancers with different p53 settings. **a** SNORD50A/B deletion frequency in 6 types of human cancers. **b** The association of SNORD50A/B deletion with poor patient survival in breast cancers (number of deletion group:188; number of wild-type group: 309), hepatocellular carcinomas (number of deletion group:28; number of wild-type group: 106) and lung adenocarcinomas (number of deletion group:21; number of wild-type group: 91). **c** The relationship between SNORD50A/B deletion and patient survival in breast cancers with mutant p53 (number of deletion group:41; number of wild-type group: 129) or wild-type p53 (number of deletion group:147; number of wild-type group: 180). **d** The relationship between p53 mutations and patient survival in breast cancers with deleted SNORD50A/B (number of mutation group:41; number of wild-type group: 147) or wild-type SNORD50A/B (number of mutation group:129; number of wild-type group: 180). All the data were from TCGA database.

We first analyzed somatic loss of the SNORD50A/B in 6 human cancer types using The Cancer Genome Atlas (TCGA) dataset. The results showed that SNORD50A/B was deleted in at least 20% of breast, ovarian, liver, and lung cancers (Fig. 1a). Moreover, SNORD50A/B deletion was strongly associated with poor patient survival in breast, liver, and lung cancers (Fig. 1b), which was consistent with a previous study [25]. By further analyzing breast cancer data from the TCGA database, we found that SNORD50A/B deletion was found in 63 of 311 (20.2%) p53mt breast cancers and 172 of 686 (25.07%) p53wt breast cancers. Besides, its deletion was significantly positively associated with poor patient survival in p53mt breast cancers, while was negatively correlated with reduced survival in p53wt breast cancers (Fig. 1c).

There is evidence showing the relationship between p53 inactivation and poor clinical outcomes of breast cancer patients [38, 39]. Thus, we analyzed the association of p53 mutations with the survival of breast cancer patients based on SNORD50A/B status. The results showed that p53 mutations were correlated with reduced survival in the patients with SNORD50A/B deletion, while almost did not affect the survival in those without SNORD50A/B deletion (Fig. 1d). Given that ER^+^HER2^-^/luminal tumors and TNBC/basal-like tumors are two major types of breast cancer with different p53 mutation status [12], we next performed the TCGA analyses separately on both ER^+^HER2^-^/luminal and TNBC/basal-like tumors. Although there was no statistical difference, SNORD50A/B deletion seems to be associated with better survival in patients with ER^+^HER2^-^/luminal tumors, while be correlated with poor survival in the patients with TNBC/basal-like tumors (Supplementary Fig. S1a). Moreover, we found that p53 mutations were significantly associated with poor survival in patients with ER^+^HER2^-^/luminal tumors, but not in those with TNBC/basal-like tumors (Supplementary Fig. S1b). Besides, we similarly analyzed the association of p53 mutations with the survival in patients with ER^+^HER2^-^/luminal tumors based on SNORD50A/B status, but not in patients with TNBC/basal-like tumors because there were only two cases simultaneously carrying wild-type p53 and SNORD50A/B deletion (Supplementary Fig. S1c). The results further supported the above conclusions, suggesting that the biological role of SNORD50A/B in breast cancer depends on p53 status.

### SNORD50A/B plays differential effects on malignant phenotypes of p53wt and p53mt breast cancer cells

**Fig. 2:**
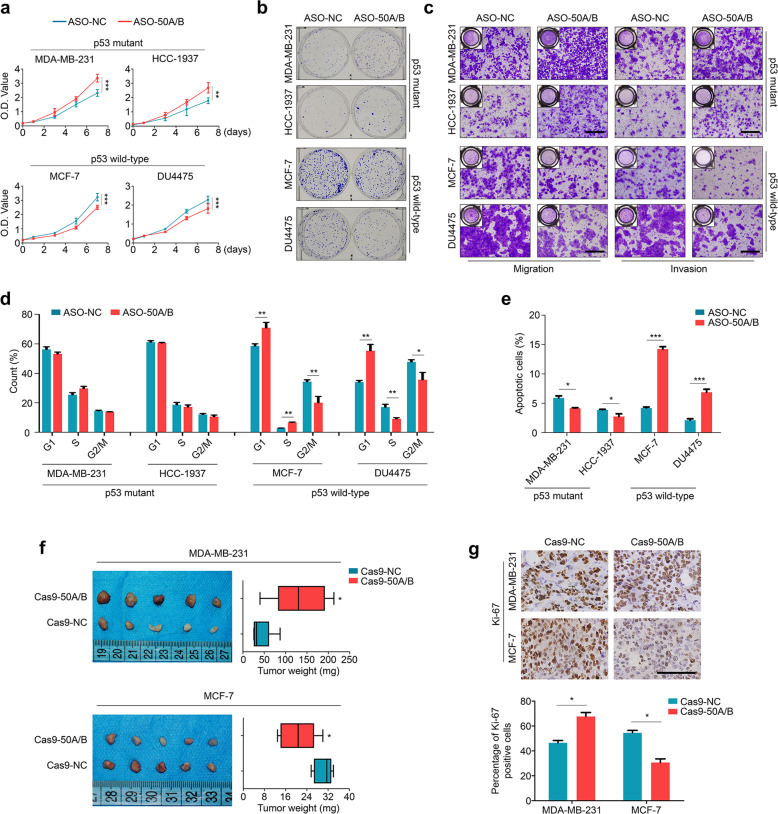
Distinct roles of SNORD50A/B knockdown or knockout in p53mt and p53wt breast cancer cells. **a** The effect of SNORD50A/B knockdown on the proliferation of the indicated cells was assessed by MTT assay. **b** The effect of SNORD50A/B knockdown on colony formation ability of the indicated cells. Shown are representative images of colony formation. **c** The effects of SNORD50A/B knockdown on migration and invasion potential of the indicated cells were evaluated by Transwell assays. Shown are representative images of migrating/invading cells. **d** The effect of SNORD50A/B knockdown on cell cycle distributions in the indicated cells. **e** The effect of SNORD50A/B knockdown on the apoptosis of the indicated cells. **f** Representative images of the indicated xenograft tumors and statistical analysis of tumor weight. **g** The levels of Ki-67 proteins in the indicated xenograft tumors by IHC assay (upper panels). Statistical analysis of the percentage of Ki-67 positive cells was shown in lower panels. Data were presented as mean ± SD. Scale bars, 200 µm. NC, negative control; 50 A/B, SNORD50A/B; *, *P* < 0.05; **, *P* < 0.01; ***, *P* < 0.001.

To verify the above speculation, we first performed qRT-PCR assay to detect the expression of SNORD50A/B in a panel of breast cancer cell lines and found that it was expressed in most of cell lines, except for T-47D cells which only expressed SNORD50B (Supplementary Fig. S2a). Next, we knocked down SNORD50A and SNORD50B in two p53mt breast cancer cell lines (MDA-MB-231 and HCC1937) and two p53wt breast cancer cell lines (MCF-7 and DU4475) by antisense oligonucleotides (ASOs) targeting SNORD50A and SNORD50B (Supplementary Fig. S2b), and found that SNORD50A and SNORD50B knockdown, either each alone or both, significantly inhibited the proliferation in p53wt breast cancer cells, while promoted the proliferation of p53mt breast cancer cells (Supplementary Fig. S3; Fig. 2a), particularly dual knockdown of SNORD50A and SNORD50B (termed “SNORD50A/B knockdown” hereafter). This was also supported by the data of colony formation (Fig. 2b; Supplementary Fig. S4), migration/invasion (Fig. 2c; Supplementary Fig. S5), and cell apoptosis (Fig. 2d). Besides, our data showed that SNORD50A/B knockdown only induced cell cycle arrest at G_0_/G_1_ phase in p53wt breast cancer cells, but not p53mt cells (Fig. 2e). On the other hand, we ectopically expressed SNORD50A/B in the above cell lines (Supplementary Fig. S6), and demonstrated that ectopic expression of SNORD50A/B inhibited the proliferation, colony formation, and migration/invasion abilities in p53mt breast cancer cells, while enhanced these malignant phenotypes in p53wt breast cancer cells (Supplementary Fig. S7a–c).

To further validate the above conclusions, we first obtained breast cancer cells stably knocking out SNORD50A/B by CRISPR-Cas9 technique (Supplementary Fig. S8), and established xenografts model in nude mice by injecting MDA-MB-231 and MCF-7 cells knocking out SNORD50A/B and control cells. The results showed that, compared to the controls, SNORD50A/B deletion significantly inhibited the growth of MCF-7 cell-derived xenograft tumors, while promoted the growth of MDA-MB-231 cell-derived xenograft tumors (Fig. 2f). This was also supported by Ki-67 staining in the above xenograft tumors (Fig. 2g).

### SNORD50A/B regulates the stability of wild-type p53 proteins via ubiquitin-proteasome degradation pathway

**Fig. 3:**
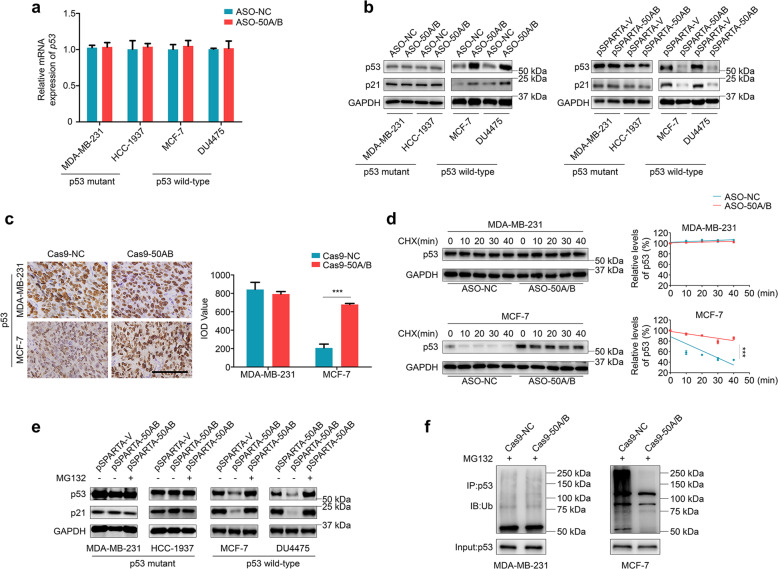
SNORD50A/B induces ubiquitin-proteasome degradation of wild-type p53. **a** The effect of SNORD50A/B knockdown on mRNA expression of *p53* in the indicated cells was evaluated by qRT-PCR. *18S* rRNA was used as a reference gene. **b** The effects of knockdown (left panel) and ectopic expression (right panel) of SNORD50A/B on protein expression of p53 and its downstream target p21 in the indicated cells were evaluated by western blot analysis. GAPDH was used as a loading control. **c** The p53 levels in in the indicated xenograft tumors were evaluated by IHC staining (left panels). Scale bars, 200 μm. The expression levels were calculated with IOD value from three different views (right panel). **d** MDA-MB-231 and MCF-7 cells were treated with 200 µg/mL CHX for the indicated times. Western blot analysis was then performed to analyze protein expression of p53 (left panels). GAPDH was used as a loading control. The band intensity of p53 in the SNORD50A/B-knockdown cells was normalized to that of GAPDH, and then normalized to that in the control cells (right panels). **e** The indicated cells were treated with 25 μM proteasome inhibitor MG132 for 3 h, and western blot analysis was then performed to evaluate the expression of p53 and its target p21. GAPDH was used as a loading control. **f** MDA-MB-231 and MCF-7 cells stably knocking out SNORD50A/B and control cells were treated with 25 μM MG132 for 2 h before harvesting. Lysates were then incubated with anti-p53 antibody and conjugated with agarose. Bounding proteins were analyzed by immunoblot with anti-ubiquitin (Ub) antibody to assess p53 ubiquitination. Data were presented as mean ± SD. ***, *P* < 0.001.

The above results suggest that there is a certain connection between biological role of SNORD50A/B and p53 proteins. Thus, we first tested the effect of SNORD50A/B knockdown on p53 expression. As shown in Fig. 3a, SNORD50A/B knockdown almost did not affect mRNA levels of p53 in both p53wt and p53mt breast cancer cells. However, compared to the controls, SNORD50A/B knockdown increased protein levels of p53 and its downstream target p21 in p53wt breast cancer cells but did not affect their protein expression in p53mt breast cancer cells (Fig. 3b, left panel). Conversely, ectopic expression of SNORD50A/B reduced protein levels of p53 and p21 in p53wt breast cancer cells, while did not affect p53 protein expression (Fig. 3b, right panel). Meanwhile, we expectedly found that ectopic expression of SNORD50A/B inhibited ERK phosphorylation in p53mt breast cancer cells, which was consistent with a previous study (Supplementary Fig. S9) [25]. Besides, we also found that SNORD50A/B knockout significantly elevated protein expression of p53 in the MCF-7 cell-derived xenograft tumors, but not in the MDA-MB-231 cell-derived xenograft tumors compared to control tumors (Fig. 3c). These data indicate that SNORD50A/B regulates the expression of wild-type p53 at post-transcriptional levels.

It is clear that ubiquitination-induced proteasomal degradation is essential for maintaining p53 protein homeostasis [15, 40]. Thus, we speculate that SNORD50A/B regulates p53 protein stability by ubiquitination-induced degradation in p53wt breast cancer cells. To verify this, we first treated breast cancer cells with CHX to block new protein synthesis and then assessed the effect of SNORD50A/B deletion on p53 protein stability. The results showed that SNORD50A/B knockdown significantly reduced the turnover of p53 proteins in p53wt breast cancer cell line MCF-7, while almost did not affect p53 protein stability in p53mt breast cancer cell line MDA-MB-231 (Fig. 3d). Next, we treated MDA-MB-231, HCC1937, MCF-7, and DU4475 cells ectopically expressing SNORD50A/B with 25 μM proteasome inhibitor MG132 for 3 h to block the ubiquitin-proteasome pathway, and found that ectopic expression of SNORD50A/B selectively inhibited protein expression of p53 and p21 in p53wt breast cancer cell lines MCF-7 and DU4475, while this effect could be reversed by MG132 treatment (Fig. 3e). In addition, we also demonstrated that SNORD50A/B deletion clearly decreased ubiquitination levels of p53 proteins in MCF-7 cells, while it had little effect on that in MDA-MB-231 cells (Fig. 3f). To further validate SNORD50A/B-mediated p53 ubiquitination, we co-expressed Ub and Flag-p53 in SNORD50A/B-deleted 293T cells and found that SNORD50A/B deletion clearly decreased ubiquitination levels of p53 proteins (Supplementary Fig. S10). These results indicate that SNORD50A/B selectively promotes the proteolysis of wild-type p53 by the ubiquitin-proteasome pathway.

### SNORD50A/B deletion stabilizes wild-type p53 proteins by promoting nuclear accumulation of GMPS

**Fig. 4:**
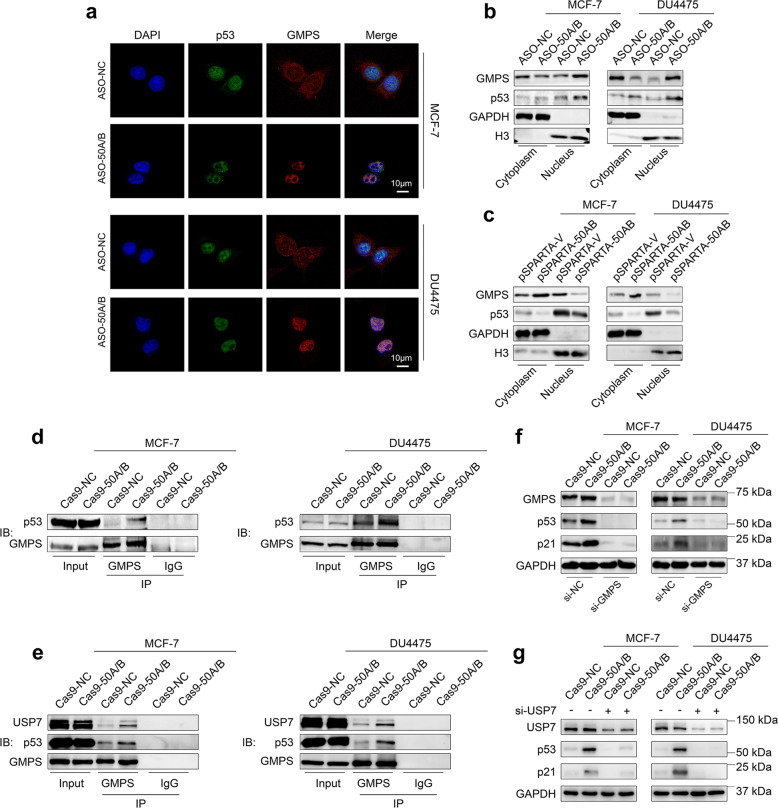
SNORD50A/B reduces p53 stability by impairing nuclear translocation of GMPS. **a** Immunofluorescence was performed to determine the translocation of GMPS and the co-localization of GMPS and p53 in MCF-7 and DU4475 cells knocking down SNORD50A/B. **b**, **c** Protein expression of GMPS and p53 in the cytoplasm and nucleus of the indicated cellsMCF-7 and DU4475 cells knocking down or ectopically expressing SNORD50A/B was evaluated by western blot analysis. GAPDH and total histone H3 were used as loading controls. **d** Cell lysates of MCF-7 and DU4475 cells knocking out SNORD50A/B were immunoprecipitated with antibodies to GMPS (mouse clonal) or IgG, and immunoblotted with antibody to p53 (rabbit clonal). **e** Immunoprecipitation was performed to verify the formation of GMPS-p53-USP7 complex in MCF-7 and DU4475 cells knocking out SNORD50A/B. MCF-7 and DU4475 cells knocking out SNORD50A/B and control cells were transfected with siRNAs targeting GMPS (**f**) or USP7 (**g**) for 48 h, western blot analysis was then performed to evaluate the expression of p53 and p21. GAPDH was used as a loading control.

A previous study has identified that TRIM21 as a potential protein binds to SNORD50A/B [25]. Moreover, TRIM21 has been indicated as a key regulator for p53 stabilization by cytoplasmic-nuclear partitioning of GMPS [41–43]. Thus, we suppose that SNORD50A/B promotes the degradation of wild-type p53 proteins by regulating TRIM21/GMPS signaling axis. To verify this, we first determined the effect of SNORD50A/B knockdown on cytoplasmic-nuclear distribution of GMPS in p53wt breast cancer cell lines MCF-7 and DU4475 by immunofluorescence assay. As shown in Fig. 4a, SNORD50A/B knockdown promoted the translocation of GMPS from the cytoplasm to the nucleus and substantially co-localized with p53 in MCF-7 and DU4475 cells. As supported, by western blot analysis, we found that SNORD50A/B knockdown increased nuclear GMPS protein levels, while decreased its cytosolic protein levels compared to the control in these two cell lines (Fig. 4b). On the contrary, ectopic expression of SNORD50A/B in these cell lines promoted cytoplasmic sequestration of GMPS compared to the control (Fig. 4c). This phenomenon also existed in p53mt breast cancer cells (Supplemental Fig. 11).

Considering that the interaction among GMPS, USP7 with p53 is required for the deubiquitination and stabilization of p53 [41, 43]. we next determined the interaction of GMPS and USP7 with p53 in the p53wt breast cancer cell lines MCF-7 and DU4475 by co-IP assays. The results showed that SNORD50A/B deletion enhanced the interaction between GMPS and p53 in these two cell lines (Fig. 4d). As a result, USP7 was recruited by GMPS, and formed a complex with GMPS and p53 [41, 42]. This was supported by our data that the interaction among GMPS, p53, and USP7 was enhanced by SNORD50A/B deletion in MCF-7 and DU4475 cells (Fig. 4e). Moreover, the interaction between p53 and USP7 was enhanced when SNORD50A/B was deleted while decreased when SNORD50A/B was ectopically expressed (Supplementary Fig. S12). Besides, protein expression of p53 and p21 was dramatically downregulated by either GMPS or USP7 knockdown in MCF-7 and DU4475 cells (Fig. 4f, g).

### SNORD50A/B induces GMPS ubiquitination and impairs its nuclear accumulation by promoting its interaction with TRIM21

**Fig. 5:**
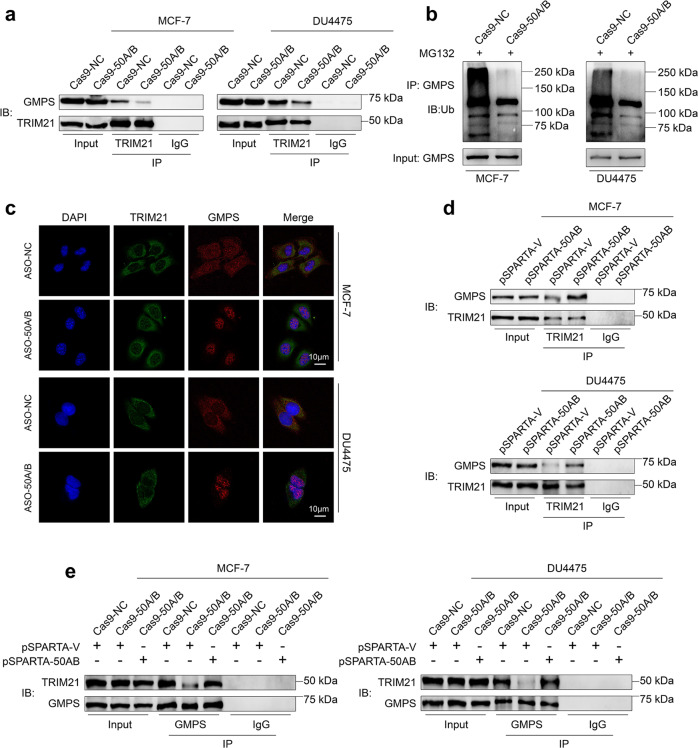
SNORD50A/B regulates ubiquitination and nuclear accumulation of GMPS by promoting its interaction with TRIM21. **a** MCF-7 and DU4475 cells stably knocking out SNORD50A/B and control cells were lysated, and the lysates were then immunoprecipitated with antibodies to TRIM21 or IgG, and immunoblotted with antibody against GMPS to validate the interaction between GMPS and TRIM21. **b** Cell lysates of indicated cells were immunoprecipitated with antibody against GMPS, and then immunoblotted with antibody against ubiquitin to evaluate GMPS ubiquitination. **c** The effect of SNORD50A/B knockdown on inducing cellular colocalization of GMPS and TRIM21 was determined by immunofluorescence assay. Blue color represents DAPI staining for nuclei; Green color represents TRIM21; Red color represents GMPS; Scale bars, 10 µm. **d** MCF-7 and DU4475 cells ectopically expressing SNORD50A/B and control cells were lysated, and the lysates then were immunoprecipitated with antibody to TRIM21 or IgG, and immunoblotted with antibody to GMPS to determine the interaction between GMPS and TRIM21. **e** The effect of restoring SNORD50A/B expression in SNORD50A/B**-**deleted MCF-7 and DU4475 cells on the interaction between GMPS and TRIM21.

There is evidence showing that TRIM21 interacts with and ubiquitylates GMPS, leading to its subcellular localization in cytoplasm [41]. Thus, we sought to investigate whether SNORD50A/B is involved in regulating the interaction between TRIM21 and GMPS, thereby affecting ubiquitination and subcellular localization of GMPS. The results showed that knocking out SNORD50A/B in MCF-7 and DU4475 cells attenuated the interaction between TRIM21 and GMPS (Fig. 5a) and decreased GMPS ubiquitination (Fig. 5b) compared to the control. Consistent with our hypothesis, SNORD50A/B deletion impaired the colocalization of TRIM21 and GMPS, and promoted the translocation of GMPS from the cytoplasm to nucleus (Fig. 5c). On the other hand, we ectopically expressed SNORD50A/B in MCF-7 and DU4475 cells, and found that ectopic expression of SNORD50A/B enhanced the interaction between TRIM21 and GMPS (Fig. 5d). Meanwhile, our results demonstrated that knocking out SNORD50A/B in MCF-7 and DU4475 cells attenuated TRIM21-GMPS interaction, while this effect could be reversed by restoring SNORD50A/B expression (Fig. 5e; Supplementary Fig. S13).

**Fig. 6:**
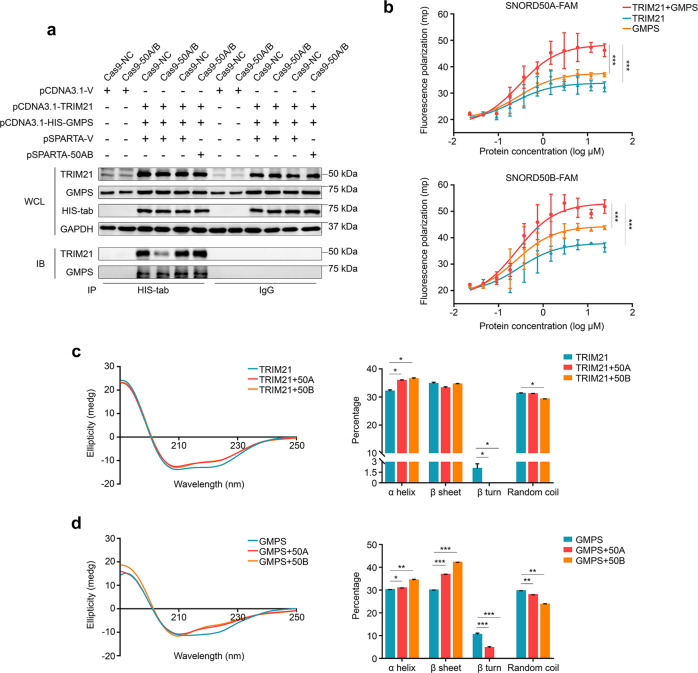
SNORD50A/B mediates the interaction between TRIM21 and GMPS. **a** HIS-GMPS and TRIM21 were co-expressed in 293 T cells with the indicated treatments, and immunoprecipitation was then performed to determine the effect of SNORD50A/B on the interaction between GMPS and TRIM21. **b** FAM-labeled SNORD50A or SNORD50B (5 μM) induces a higher polarization of fluorescence when mixed with the combination of TRIM21 and GMPS than each alone. The effect of SNORD50A or SNORD50B on secondary structure of TRIM21 (**c**) and GMPS (**d**) proteins. Data were presented as mean ± SD. *, *P* < 0.05; **, *P* < 0.01; ***, *P* < 0.001.

To further validate SNORD50A/B-mediated TRIM21-GMPS interaction, we co-expressed His-GMPS, TRIM21, and Ub or SNORD50A/B in 293T cells (Supplementary Fig. S14a, b). The results showed that SNORD50A/B deletion clearly decreased GMPS ubiquitination (Supplementary Fig. S15). Moreover, SNORD50A/B deletion diminished TRIM21-GMPS interaction, and this effect was restored by re-expressing SNORD50A/B in SNORD50A/B-deleted 293 T cells (Fig. 6a). These data indicate that SNORD50A/B is involved in regulating the interaction between TRIM21 and GMPS. Next, we attempted to clarify the exact mechanism. A previous study has shown a potential interaction between SNORD50A/B and TRIM21 [25, 44]. Thus, we speculate that SNORD50A/B may bind to TRIM21, promoting its interaction with GMPS. To prove this, recombinant TRIM21 and GMPS proteins were expressed, purified, and confirmed by HPLC (Supplementary Fig. S16a, b).

Fluorescence polarization technique has been widely used to detect the interaction among molecules [45, 46]. Thus, in this study, we used this technique to detect the interaction between FAM-labeled SNORD50A or SNORD50B and TRIM21 or GMPS. The results showed that FAM-labeled SNORD50A or SNORD50B induced an increased fluorescence polarization when mixed with sequentially diluted TRIM21 or GMPS (Fig. 6b), while FAM-labeled scrambled RNA sequence (as a negative control) almost did not affect fluorescence polarization (Supplementary Fig. S17). These results indicate that SNORD50A/B can bind directly to TRIM21 or GMPS. It should be noted that FAM-labeled SNORD50A or SNORD50B induced a higher fluorescence polarization when mixed SNORD50A/B with TRIM21 and GMPS (Fig. 6b), indicating that SNORD50A/B forms a complex with TRIM21 and GMPS, thereby enhancing TRIM21-GMPS interaction.

Circular dichroism (CD) spectroscopy is generally used as a tool to study protein secondary structure [47, 48]. In this study, we examined the conformation of TRIM21 and GMPS interacting with SNORD50A/B using CD spectroscopy. The results showed that SNORD50A/B-TRIM21 interaction significantly increased α-Helix content, while decreased β-turn and random coil content of TRIM21 (Fig. 6c). Conformational conversion of GMPS was triggered by SNORD50A/B with an increase in α-Helix and β-sheet content, and decrease in β-turn and random coil content (Fig. 6d). These data further support direct interaction of SNORD50A/B with TRIM21 and GMPS.

**Fig. 7:**
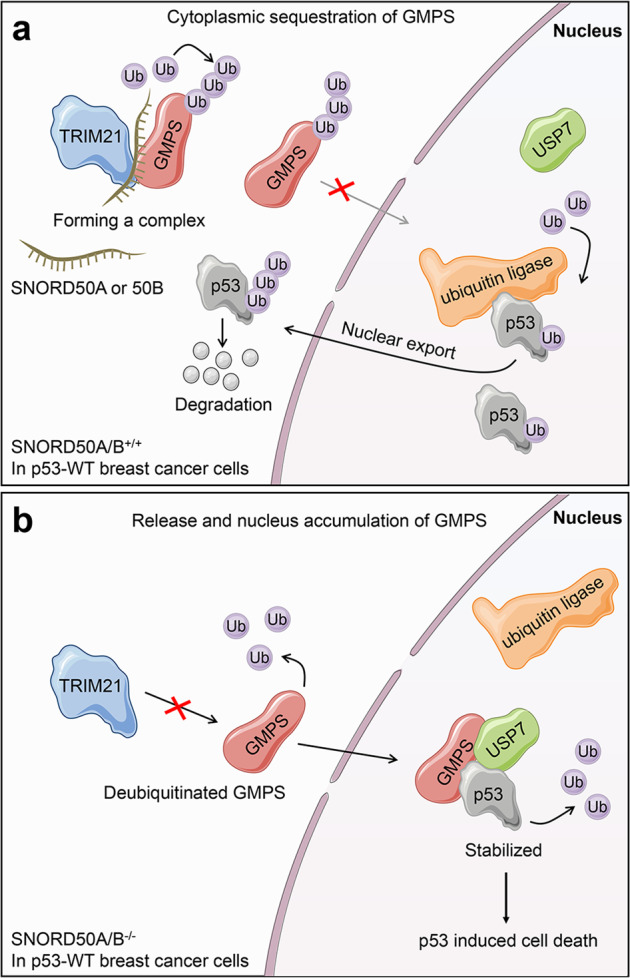
A schematic model of SNORD50A/B inducing ubiquitin-proteasome degradation of wild-type p53 by promoting the interaction between TRIM21 and GMPS. **a** In general, in p53wt breast cancer cells, SNORD50A/B promotes the interaction between TRIM21 and GMPS by directly binding them. As a result, GMPS is ubiquitinated by TRIM21 and sequestered in the cytoplasm, thereby leading to p53 ubiquitination and degradation. **b** In SNORD50A/B-deleted p53wt breast cancer cells, GMPS can be released into the nucleus, where GMPS can recruit USP7 and form a complex with p53, thereby decreasing p53 ubiquitination and increasing its protein stability.

Based on the above observations, we propose a simple model to illustrate the mechanism of SNORD50A/B promoting the growth of p53wt breast cancers (Fig. 7). SNORD50A/B forms a complex with TRIM21 and GMPS in the cytoplasm, leading to ubiquitylation and cytoplasmic sequestration of GMPS by TRIM21. This will cause ubiquitylation, nuclear exporting, and subsequent degradation of p53, as reviewed in a previous study [49]. The degradation of p53 then promotes the growth of p53wt breast cancers (Fig. 7a). However, in SNORD50A/B-deleted p53wt breast cancer cells, GMPS is released into the nucleus and forms a complex with p53 and USP7, leading to deubiquitylation and stabilization of p53 (Fig. 7b).

## Discussion

The location of small nucleolar RNA SNORD50A/B was identified as a breakpoint in human B-cell lymphoma, which is the first study that connected SNORD50A/B to tumorigenesis [50]. SNORD50A/B has been demonstrated to be highly deleted in different types of cancer and exerts its tumor suppressor function by inhibiting the activity of KRAS oncoproteins [24, 25]. However, the present study surprisingly found that biological role of SNORD50A/B was dependent on p53 status in breast cancers. Briefly, our data showed that SNORD50A/B knockdown significantly inhibited malignant phenotypes of p53wt breast cancer cells, such as the inhibition of cell proliferation, migration, invasion and tumorigenic potential, and the induction of cell cycle arrest and apoptosis, while knocking down SNORD50A/B in p53mt breast cancer cells showed an opposite effect on these malignant behaviors. On the other hand, ectopic expression of SNORD50A/B further supported the above conclusions. These results indicate that SNORD50A/B may play oncogenic functions in p53wt breast cancers.

p53 acts as a key tumor suppressor in breast cancers and its inactivation will accelerate tumor progression [8, 51]. Mutations of the *p53* gene are a major cause of its inactivation [52, 53]. In addition, aberrant activation of ubiquitin–proteasome system is also critical for p53 inactivation by inducing its degradation [14, 54]. Our data showed that ectopic expression of SNORD50A/B in p53wt breast cancer cells dramatically reduced the expression of p53 proteins, while SNORD50A/B knockdown increased p53 protein stability by inhibiting its ubiquitination degradation. Thus, we conclude that SNORD50A/B promotes malignant phenotypes of p53wt breast cancer cells by inducing p53 ubiquitination and degradation.

Next, we attempted to explore the mechanism of SNORD50A/B regulating p53 ubiquitination in p53wt breast cancer cells. A previous study has identified that TRIM21 as a potential protein binds to SNORD50A/B [25]. Moreover, the TRIM2-GMPS-USP7 connection has been reported as a shared molecular mechanism that stabilizes wild-type p53 proteins, but not mutated p53 proteins [41–43]. As supported, we demonstrated that SNORD50A/B promoted p53 ubiquitination and degradation via the TRIM2-GMPS-USP7 signaling axis in p53wt breast cancer cells. In brief, SNORD50A/B strengthened the interaction between TRIM21 and GMPS, thereby ubiquitinating and sequestering GMPS in the cytoplasm; however, SNORD50A/B deletion significantly attenuated the interaction between them, leading to GMPS deubiquitylation. Deubiquitinated GMPS moves to the nucleus and recruits deubiquitinating enzyme USP7, forming a complex with and stabilizing p53. To be consistent with these observations, USP7 has been indicated to act as a tumor suppressor in breast cancers by activating the p53 pathway [55, 56]. In addition, our data showed that SNORD50A/B deletion induced the translocation of GMPS not only in p53wt breast cancer cells but also in p53mt breast cancer cells. However, SNORD50A/B deletion almost did not affect the stability and expression of mutated p53 proteins, but activated the KRAS/RAF/MEK/ERK pathway in p53mt breast cancer cells.

In this study, we demonstrated that SNORD50A/B could directly bind to TRIM21 by fluorescence polarization technique, as supported by the previous studies [25, 44]. Notably, our data, for the first time, showed that SNORD50A/B could also directly bind to GMPS. Besides, by using CD spectroscopy, we revealed a conversion of the secondary conformation in these two proteins when binding to SNORD50A/B. These findings suggest that conformational conversion of TRIM21 and GMPS may contribute to their interaction. However, further structural analysis is needed to validate this conclusion.

In summary, our data find that SNORD50A/B plays differential roles in p53wt and p53mt breast cancer cells. Similar to the previous studies [24, 57], SNORD50A/B functions as a tumor suppressor in p53mt breast cancer cells, while play oncogenic functions in p53wt breast cancer cells by mediating GMPS-TRIM21 interaction and subsequently inducing ubiquitin-proteasome degradation of p53. In this study, our data show that SNORD50A/B can directly bind to both TRIM21 and GMPS, and induce their conformational conversion. This will be crucial for the interaction between them. Thus, unique features of our finding may offer some specific opportunities for mechanism exploring and drug development by targeting SNORD50A/B in p53wt breast cancers.

## Supplementary information

Supplementary tables and figure legends

Figure S1

Figure S2

Figure S3

Figure S4

Figure S5

Figure S6

Figure S7

Figure S8

Figure S9

Figure S10

Figure S11

Figure S12

Figure S13

Figure S14

Figure S15

Figure S16

Figure S17
