# Supplementary material for: The noncoding RNAs SNORD50A and SNORD50B-mediated TRIM21-GMPS interaction promotes the growth of p53 wild-type breast cancers by degrading p53
Source: Cell Death Differ. 2021 Mar 19;28(8):2450–64. doi: 10.1038/s41418-021-00762-7 (PMC8329294; doi:10.1038/s41418-021-00762-7)
Supplement: Supplementary file 1 — Supplementary tables and figure legends [file 41418_2021_762_MOESM1_ESM.docx]

**Supplemental Information**

**The noncoding RNAs SNORD50A and SNORD 50B-mediated TRIM21-GMPS interaction promotes the growth of p53 wild-type breast cancers by degrading p53**

Xi Su^1^, Chao Feng^1^, Simeng Wang^1^, Liang Shi^1^, Qingqing Gu^1^, Haihong Zhang^1^, Xinhui Lan^1^, Yuelei Zhao^1^, Wei Qiang^1^, Meiju Ji^2, *^, and Peng Hou^1, *^

^1^ Key Laboratory for Tumor Precision Medicine of Shaanxi Province and Department of Endocrinology, The First Affiliated Hospital of Xi’an Jiaotong University, Xi’an 710061, P.R. China

^2^ Center for Translational Medicine, The First Affiliated Hospital of Xi’an Jiaotong University, Xi’an 710061, P.R. China

**Running title**

Oncogenic role of SNORD50A/B in p53 wild-type breast cancers

**Conflict of Interest**

The authors declare that they have no conflict of interest.

***To whom correspondence should be addressed**

**Peng Hou, Ph.D.** Key Laboratory for Tumor Precision Medicine of Shaanxi Province and Department of Endocrinology, The First Affiliated Hospital of Xi’an Jiaotong University, Xi’an 710061, P.R. China. Tel/Fax: +86 29 8532 4749; E-mail: [phou@xjtu.edu.cn](mailto:phou@xjtu.edu.cn)

**Meiju Ji, Ph.D.** Center for Translational Medicine, The First Affiliated Hospital of Xi’an Jiaotong University, Xi’an 710061, P.R. China. Tel/Fax: +86 29 8532 3259; E-mail: [mjji0409@163.com](mailto:mjji0409@163.com)

**Supplemental Data**

**Supplemental Table 1**

Sequences used in this study

**Supplemental Table 2**

The primers used in this study

**Supplemental Table 3**

The antibodies used in this study

**Supplemental Fig. 1**

**a** The relationship between SNORD50A/B deletion and patient survival in ER+/HER2- Breast cancers (number of deletion group:118; number of wild-type group: 191) or TNBC (number of deletion group:11; number of wild-type group: 79). **b** The relationship between p53 mutation and patient survival in ER+/HER2- Breast cancers (number of p53 wild-type group:380; number of p53 mutant group: 77) or TNBC (number of p53 wild-type group:33; number of p53 mutant group: 90). **c** The relationship between SNORD50A/B deletion and patient survival in ER+/HER2- Breast cancers with mutant p53 (number of deletion group:18; number of wild-type group: 43) or wild-type p53 (number of deletion group:100; number of wild-type group: 148).

**Supplemental Fig.2**

**a** SNORD50A/B expression in a panel of breast cancer cells was determined by RT-PCR assay. *U6* was used as a reference gene. **b** The qRT-PCR assays of SNORD50A/B to show their knockdown efficiency in MDA-MB-231, HCC1937, MCF-7 and DU4475 cells. *U6* was used as a reference gene. All the experiments were carried out in triplicate. Data were presented as mean ± SD. ***, *P* < 0.001.

**Supplemental Fig.3**

The effect of SNORD50A or SNORD50B knockdown on the proliferation of the indicated cells were examined by MTT assay. Data were presented as mean ± SD. *, *P* <0.05; **, *P* <0.01; ***, *P* <0.001 for the comparison between SNORD50A knockdown and the control; ^#^, *P* <0.05; ^##^, *P* <0.01; *^###^*, *P* <0.001 for the comparison between SNORD50B knockdown and the control.

**Supplemental Fig.4**

Quantitative analysis of colony formation in MDA-MB-231, HCC1937, MCF-7 and DU4475 cells with the indicated treatments. Data were presented as mean ± SD. ***, *P* <0.001.

**Supplemental Fig.5**

Quantitative analysis of migrating cells (upper panel) and invading cells (lower panel) in MDA-MB-231, HCC1937, MCF-7 and DU4475 cells with the indicated treatments. Data were presented as mean ± SD. *, *P* <0.05; **, *P* <0.01; ***, *P* <0.001.

**Supplemental Fig.6**

qRT-PCR assays of SNORD50A/B to show their ectopic expression efficiency in MDA-MB-231, HCC1937, MCF-7 and DU4475 cells. *U6* was used as a reference gene. All the experiments were carried out in triplicate. Data were presented as mean ± SD. ***, *P* < 0.001.

**Supplemental Fig.7**

**a** The effect of ectopic expression of SNORD50A/B on the proliferation of the indicated cells were examined by MTT assay. **b** Representative images of colony formation in the indicated cells (left panels). Quantitative analysis was shown in right panels. **c** Representative images of migrating/invading cells in the indicated cells (left panels). Quantitative analysis was shown in right panels. All the experiments were carried out in triplicate. Data were presented as mean ± SD. Scale bars, 200 µm. *, *P* <0.05; **, *P* <0.01; ***, *P* <0.001.

**Supplemental Fig.8**

qRT-PCR assays of SNORD50A/B to show their knockout efficiency in MDA-MB-231, HCC1937, MCF-7 and DU4475 cells. *U6* was used as a reference gene. All the experiments were carried out in triplicate. Data were presented as mean ± SD. ***, *P* < 0.001.

**Supplemental Fig.9**

The effects of ectopic expression of SNORD50A/B on protein expression of p53 and ERK phosphorylation in the indicated cells were evaluated by western blot analysis. GAPDH was used as a loading control.

**Supplemental Fig.10**

Flag-p53 and Ub were co-expressed in 293T cells with MG132, and immunoprecipitation was then performed to determine the effect of SNORD50A/B on the p53 ubiquitination.

**Supplemental Fig.11**

Protein expression of GMPS and p53 in the cytoplasm and nucleus of MDA-MB-231 and HCC1937 cells was evaluated by western blot analysis. GAPDH and total histone H3 were used as loading controls.

**Supplemental Fig.12**

MCF-7 cells knocking out or ectopic expression SNORD50A/B were lysed. Immunoprecipitation was performed to verify the combination of p53 and USP7 in the cells.

**Supplemental Fig.13**

qRT-PCR was performed to determine the restoration of SNORD50A/B in SNORD50A/B-deleted MCF-7 and DU4475 cells. All the experiments were carried out in triplicate. Data were presented as mean ± SD. ***, P < 0.001.

**Supplemental Fig.14**

**a** qRT-PCR was performed to determine the restoration of SNORD50A/B in **a** qRT-PCR was performed to determine the restoration of SNORD50A/B in SNORD50A/B-deleted 293T cells. **b** Western blot analysis was performed to determine successful co-transfection of TRIM21 and GMPS in 293T cells with the indicated treatments. All the experiments were carried out in triplicate. Data were presented as mean ± SD. ***, *P* < 0.001.

**Supplemental Fig.15**

His-GMPS, TRIM21 and Ub were co-expressed in 293T cells, then cells were treated with MG132 and immunoprecipitation was performed to determine the effect of SNORD50A/B deletion on the GMPS ubiquitination.

**Supplemental Fig.16**

**a** Coomassie Blue staining was performed to prove successful expression of TRIM21 and GMPS. **b** Purified TRIM21 and GMPS were confirmed by HPLC. Dotted boxes indicate TRIM21 and GMPS proteins.

**Supplemental Fig.17**

Fluorescence Polarization assay was performed to determine the interaction of scrambled RNA (negative control) with TRIM21 or/and GMPS.

**Supplemental Table 1.** Sequences used in this study

| **ASOs/siRNAs** | **5’- Sequence -3’** |
| --- | --- |
| ASO-SNORD50A | zCxzUxzCxzAxzGxAxAxGxCxCxAxGxAxTxCxzCxzGxzUxzAxzA |
| ASO-SNORD50B | zCxzUxzCxzAxzGxAxAxGxCxCxGxAxAxTxCxzCxzGxzUxzAxzC |
| ASO-NC | zUxzCxzAxzCxzCxTxTxCxAxCxCxCxTxCxTxzCxzCxzAxzCxzU |
| si-GMPS-1 | ACUUUGUCUACACUAUCUC |
| si-GMPS-2 | AAACUGUUGAGUCUACUCC |
| si-USP7-1 | UAUUUGAUAACGUAACCACTT |
| si-USP7-2 | UUAUUAUCUUCAGCACUGCTT |

All ASOs used for targeting snoRNAs were chemically synthesized as 5-10-5 RNA/DNA chimeric oligonucleotides linked with phosphorothioate backbones, with 10 deoxyribonucleotides flanked by 5 2’-O-MOE modified ribonucleotides at both sides (z–2’-O-methoxyethyl, x-phosphorothioate backbone).

**Supplemental Table 2.** The primers used in this study

| **Primers** | **Forward (5’-3’)** | **Reverse (5’-3’)** |
| --- | --- | --- |
| *SNORD50A* | TATCTGTGATGATCTTATCCCGAACCTGAAC | ATCTCAGAAGCCAGATCCGTAA |
| *SNORD50B* | TAATCAATGATGAAACCTATCCCG | TAATCTCAGAAGCCGAATCCGTA |
| *U6* | GCTTCGGCAGCACATATACTAAAAT | CGCTTCACGAATTTGCGTGTCAT |
| *p53* | CCTCAGCATCTTATCCGAGTGG | TGGATGGTGGTACAGTCAGAGC |
| *GMPS* | CCCATCACAATGACACAGAGCTC | CTGGAAGTCCAAGTTCTCTGCC |
| *18S* | CGCCGCTAGAGGTGAAATTC | CTTTCGCTCTGGTCCGTCTT |

| **Antibodies** | **species** | **Catalog#** | **Source** |
| --- | --- | --- | --- |
| anti-Ubiquitin | R | Ab7780 | Abcam |
| anti-Flag tag | R | ab236777 | Abcam |
| anti-Ki67 | M | 550609 | BD Pharmingen |
| anti-GAPDH | R | AP0063 | Bioworld Technology |
| anti-p21 | R | 2947s | Cell Signaling Technology |
| anti-p53 | R | 9282s | Cell Signaling Technology |
| anti-TRIM21 | R | 92043s | Cell Signaling Technology |
| anti-USP7 | R | 4833S | Cell Signaling Technology |
| anti-Ubiquitin | M | sc-8017 | Santa Cruz |
| anti-USP7 | M | sc-137008 | Santa Cruz |
| anti-GMPS | M | sc-376163 | Santa Cruz |
| anti-p53 | M | sc-126 | Santa Cruz |
| anti-TRIM21 | M | sc-25351 | Santa Cruz |
| anti-HIS tag | R | sc-803 | Santa Cruz |

**Supplemental Table 3.** The antibodies used in this study

R: rabbit; M: mouse
