# Supplementary figures and images for: The noncoding RNAs SNORD50A and SNORD50B-mediated TRIM21-GMPS interaction promotes the growth of p53 wild-type breast cancers by degrading p53
Source: Cell Death Differ. 2021 Mar 19;28(8):2450–64. doi: 10.1038/s41418-021-00762-7 (PMC8329294; doi:10.1038/s41418-021-00762-7)

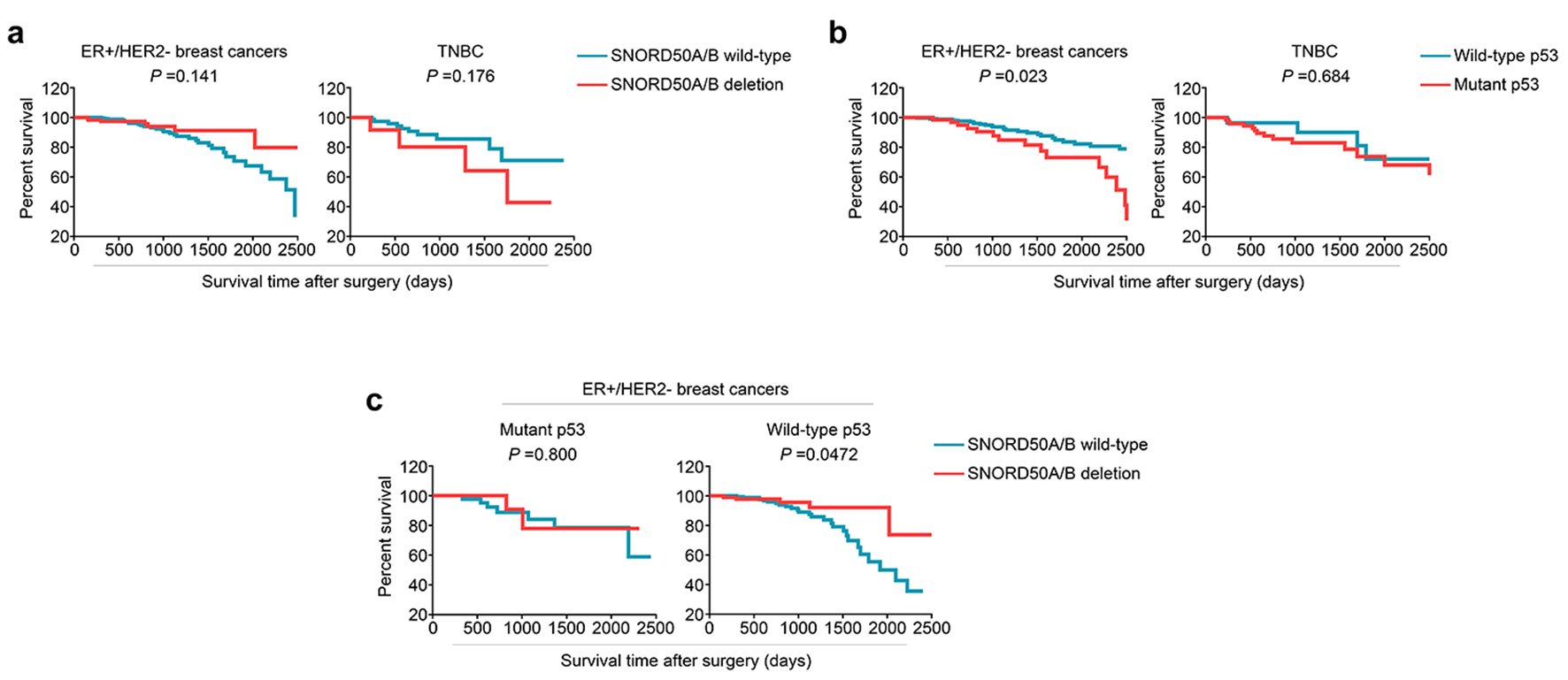

Supplement: Supplementary file 2 — Figure S1 [file 41418_2021_762_MOESM2_ESM.tif]

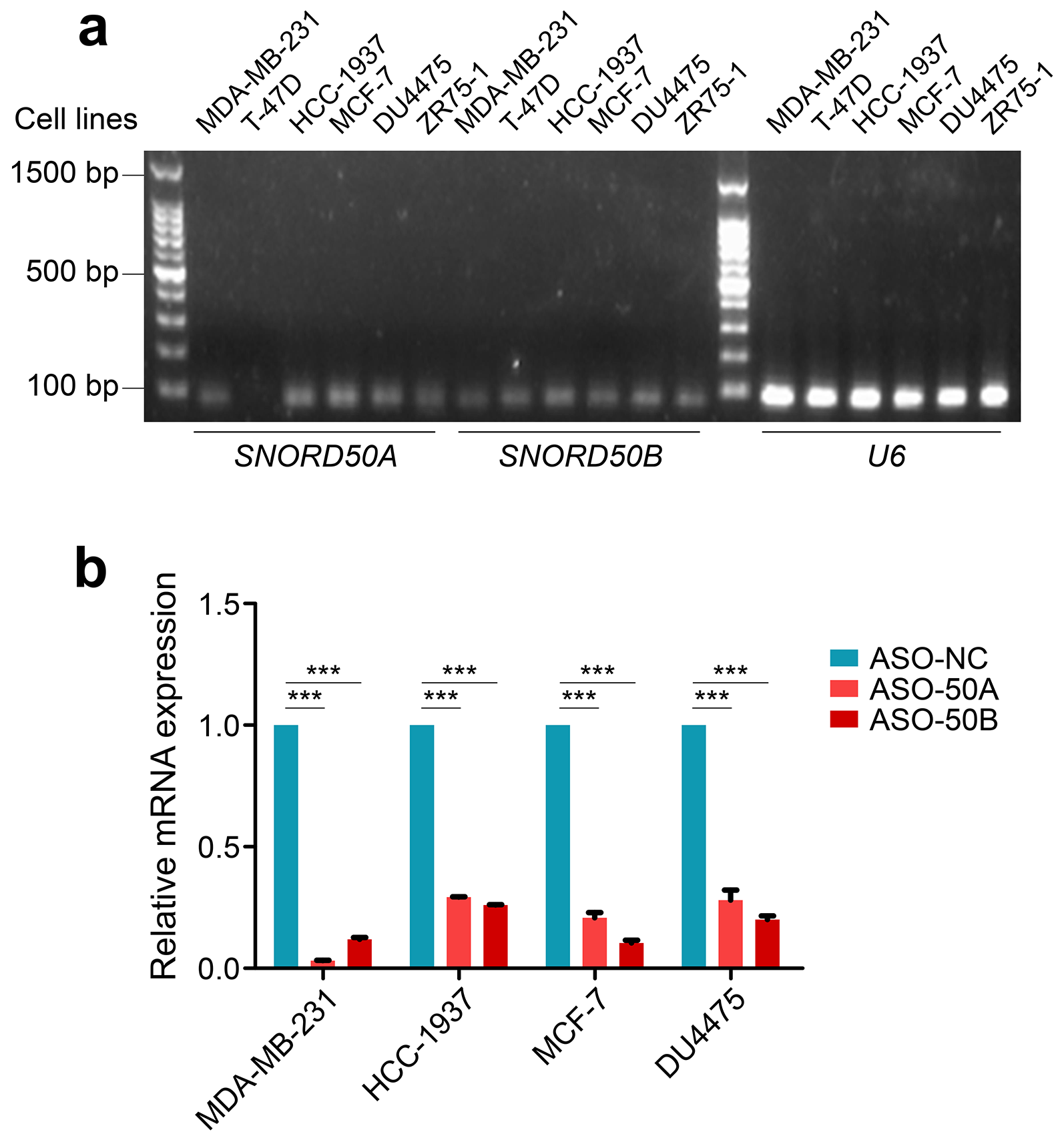

Supplement: Supplementary file 3 — Figure S2 [file 41418_2021_762_MOESM3_ESM.tif]

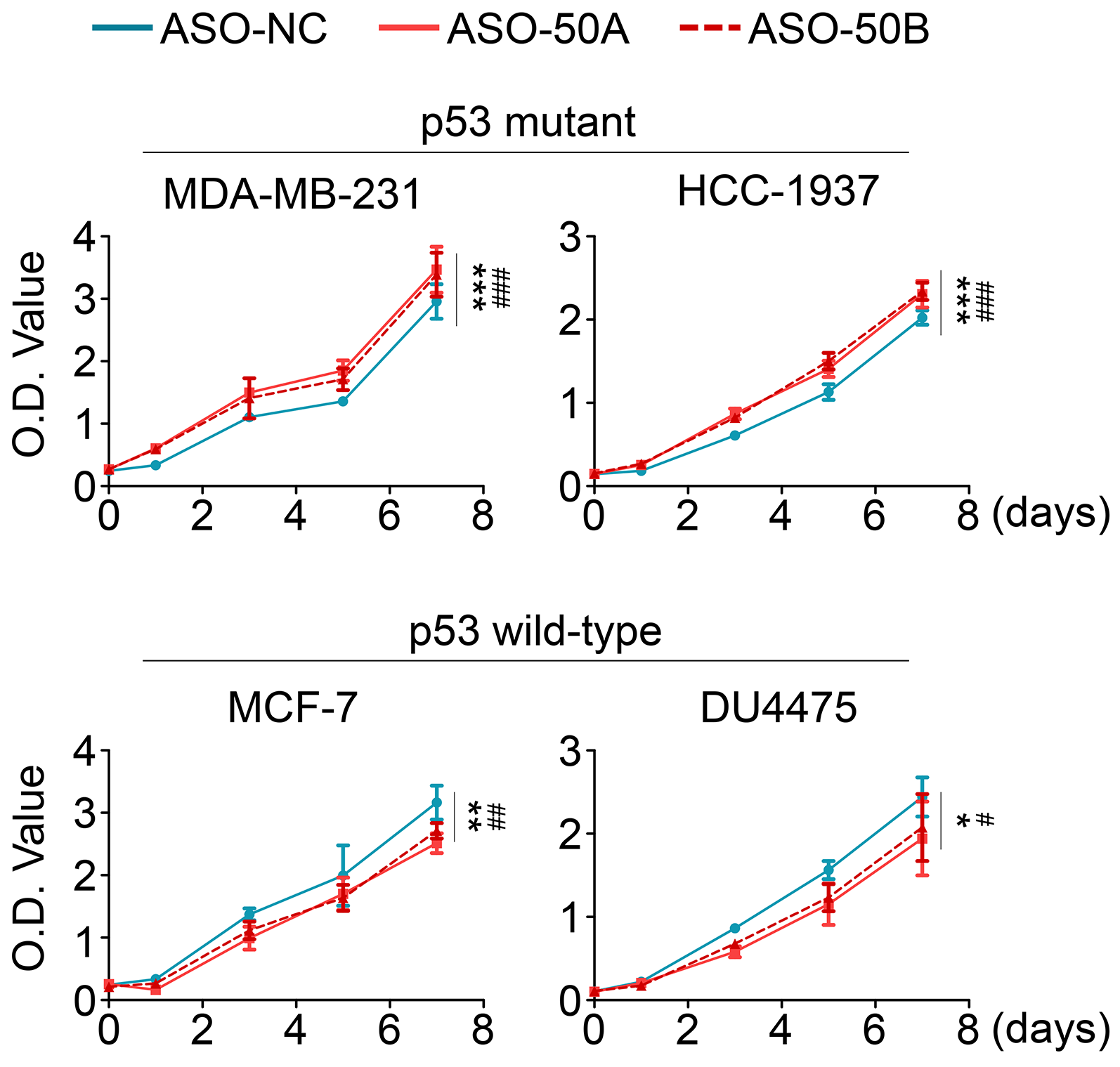

Supplement: Supplementary file 4 — Figure S3 [file 41418_2021_762_MOESM4_ESM.tif]

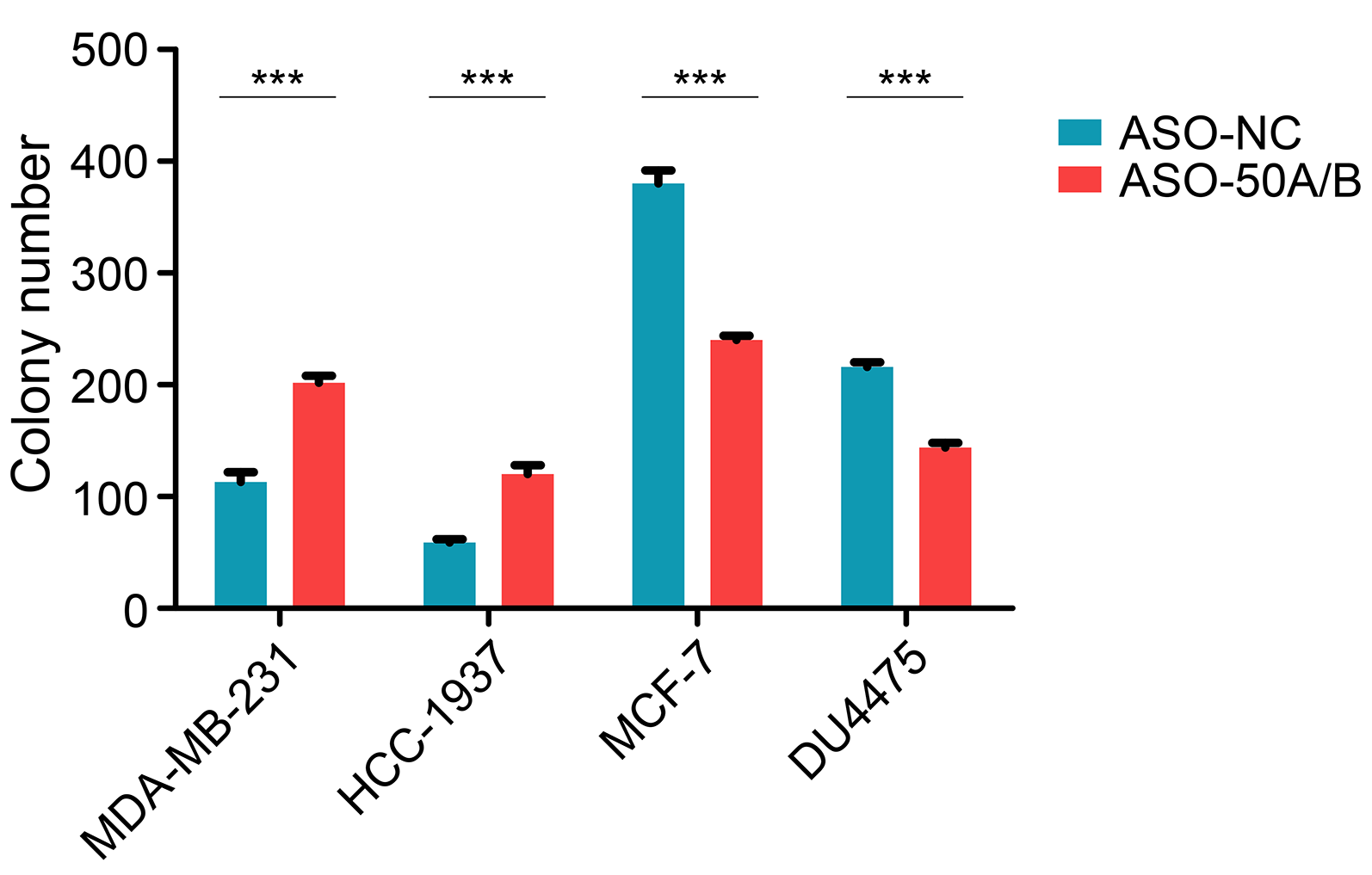

Supplement: Supplementary file 5 — Figure S4 [file 41418_2021_762_MOESM5_ESM.tif]

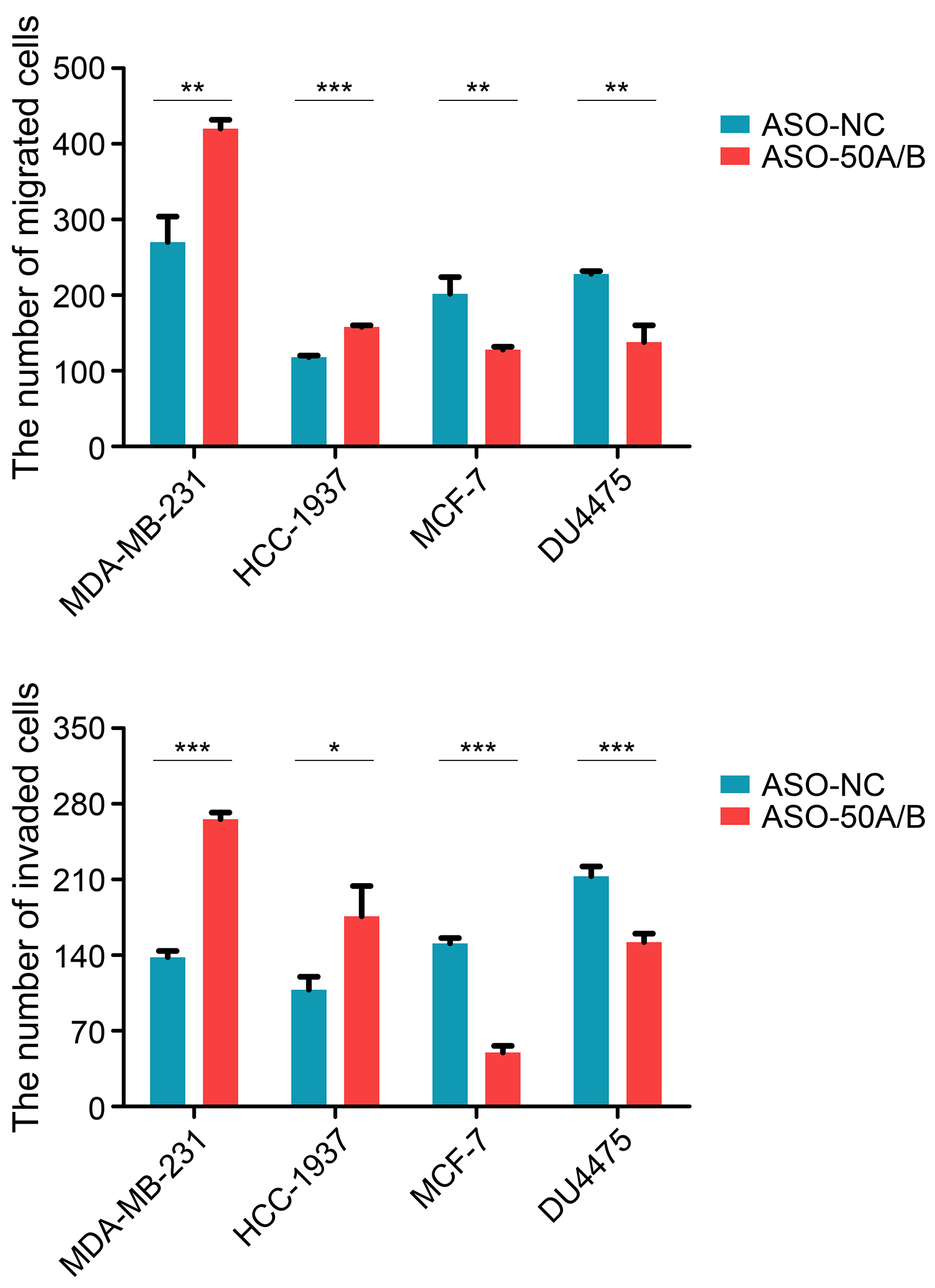

Supplement: Supplementary file 6 — Figure S5 [file 41418_2021_762_MOESM6_ESM.tif]

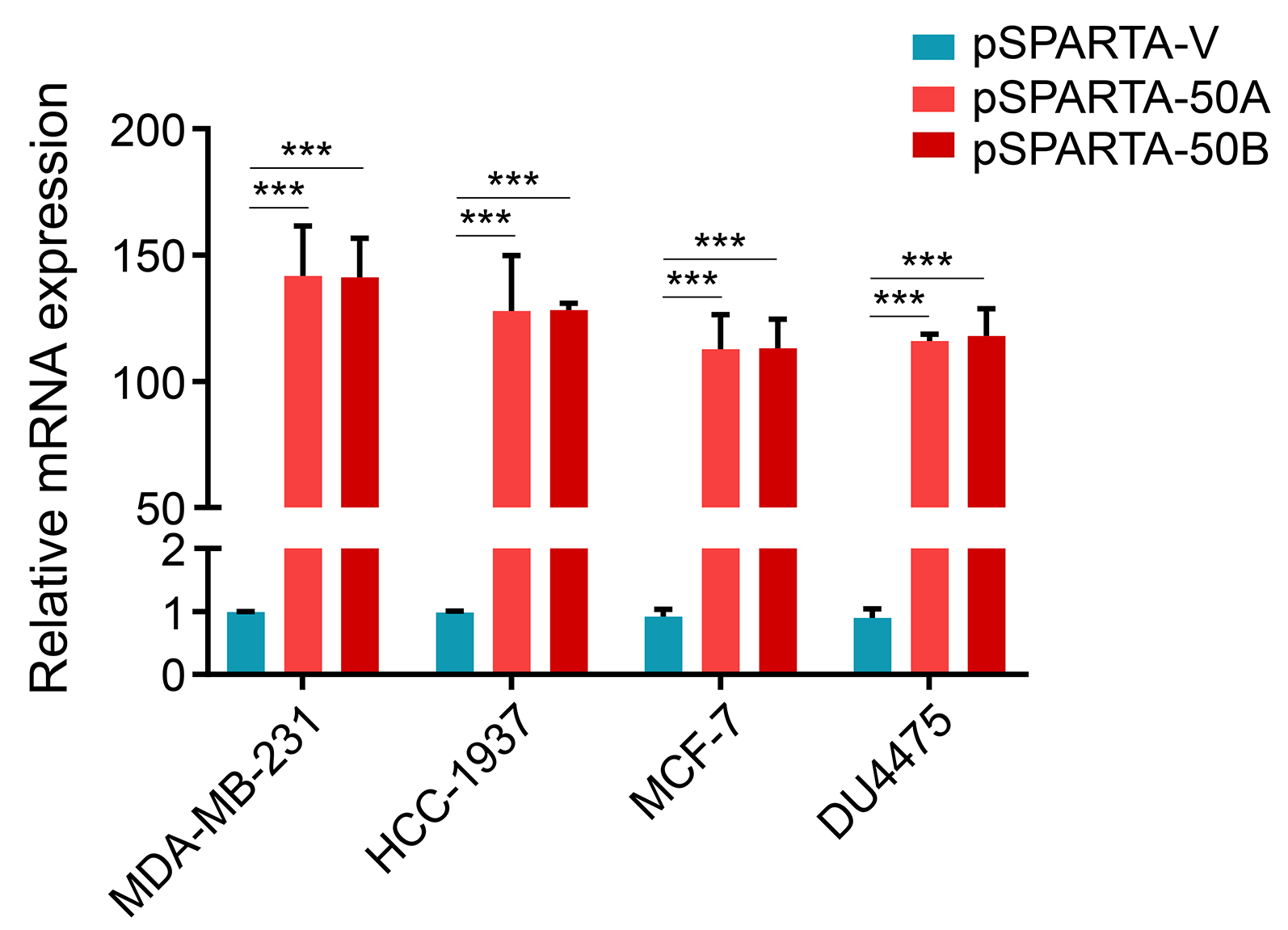

Supplement: Supplementary file 7 — Figure S6 [file 41418_2021_762_MOESM7_ESM.tif]

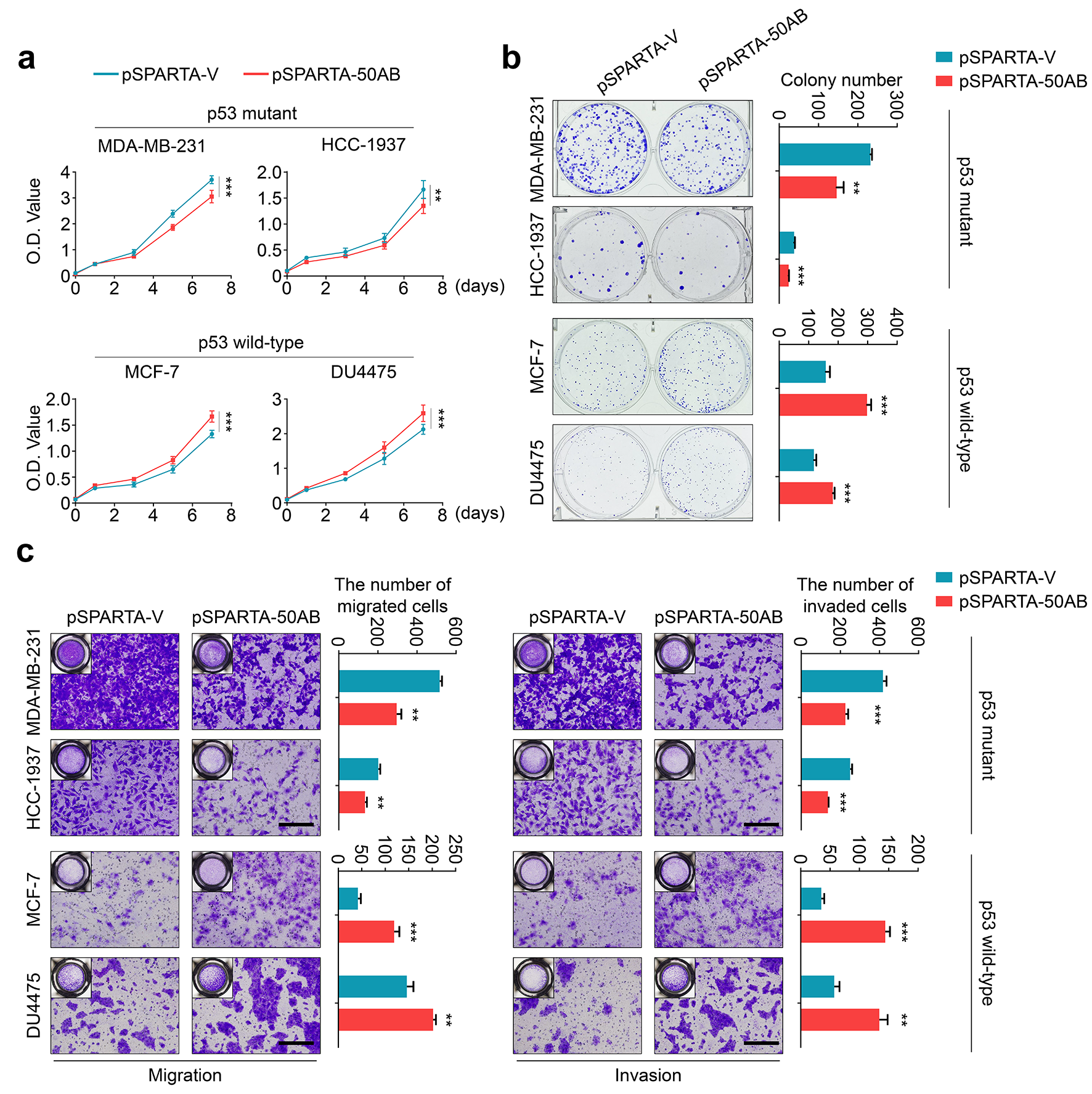

Supplement: Supplementary file 8 — Figure S7 [file 41418_2021_762_MOESM8_ESM.tif]

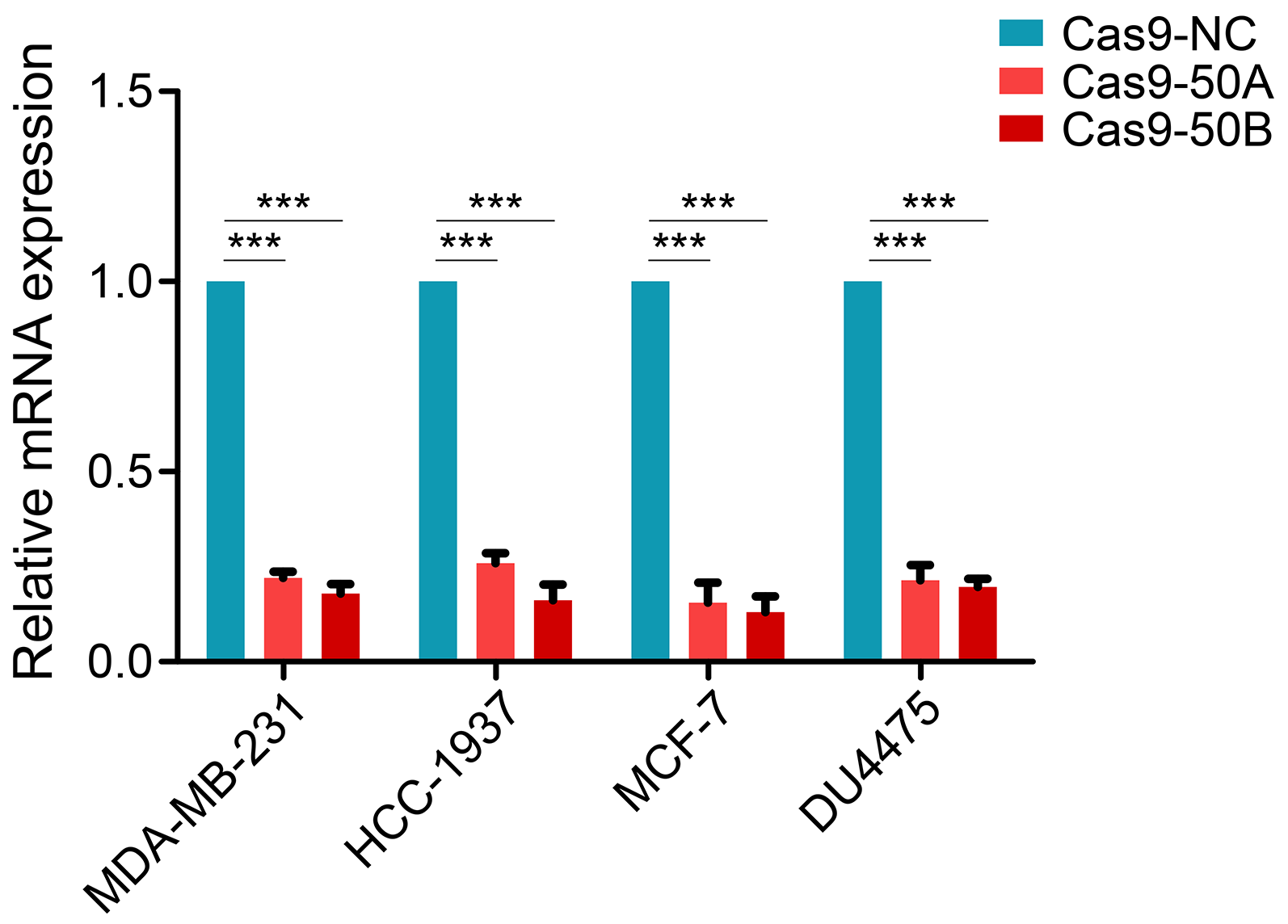

Supplement: Supplementary file 9 — Figure S8 [file 41418_2021_762_MOESM9_ESM.tif]

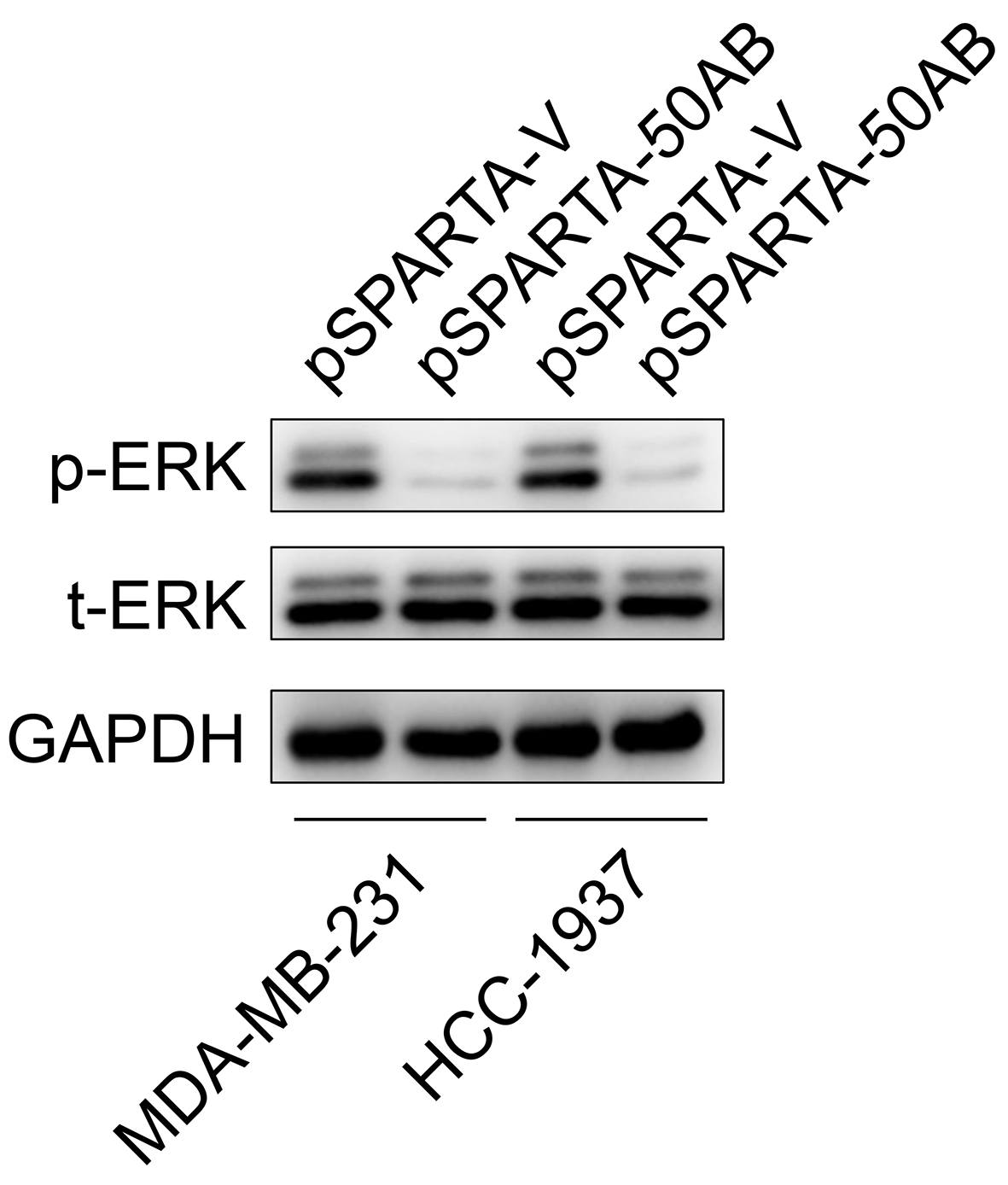

Supplement: Supplementary file 10 — Figure S9 [file 41418_2021_762_MOESM10_ESM.tif]

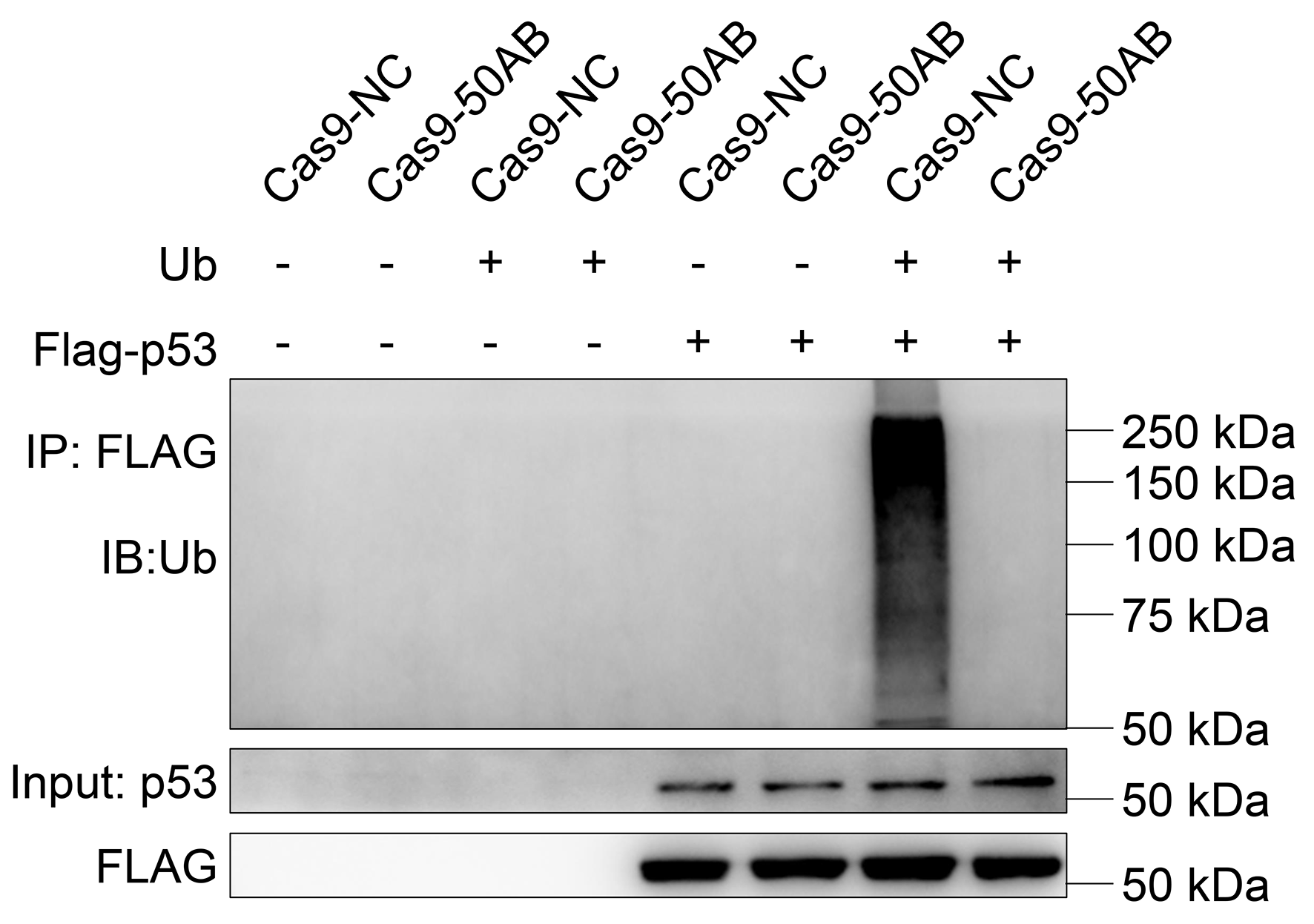

Supplement: Supplementary file 11 — Figure S10 [file 41418_2021_762_MOESM11_ESM.tif]

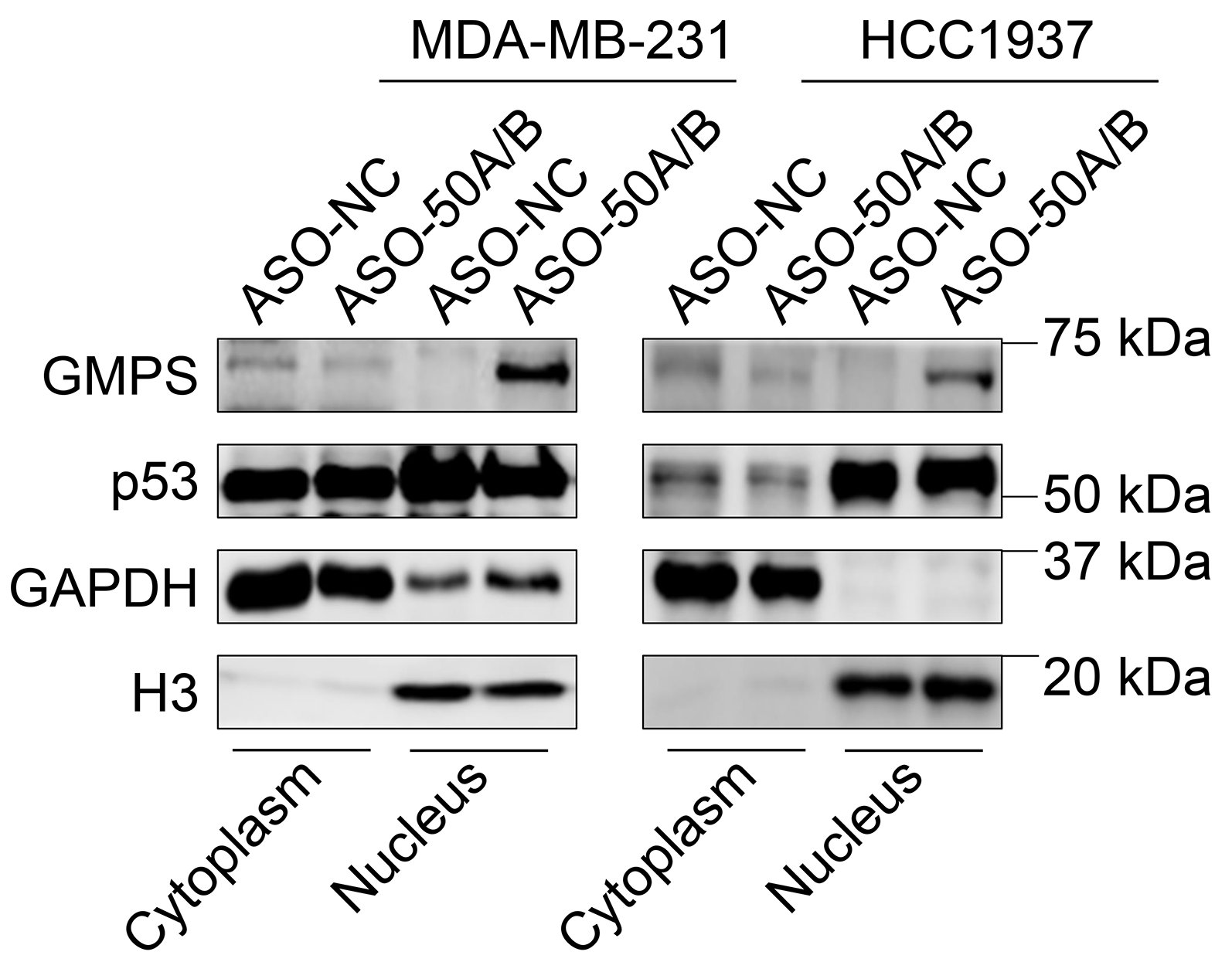

Supplement: Supplementary file 12 — Figure S11 [file 41418_2021_762_MOESM12_ESM.tif]

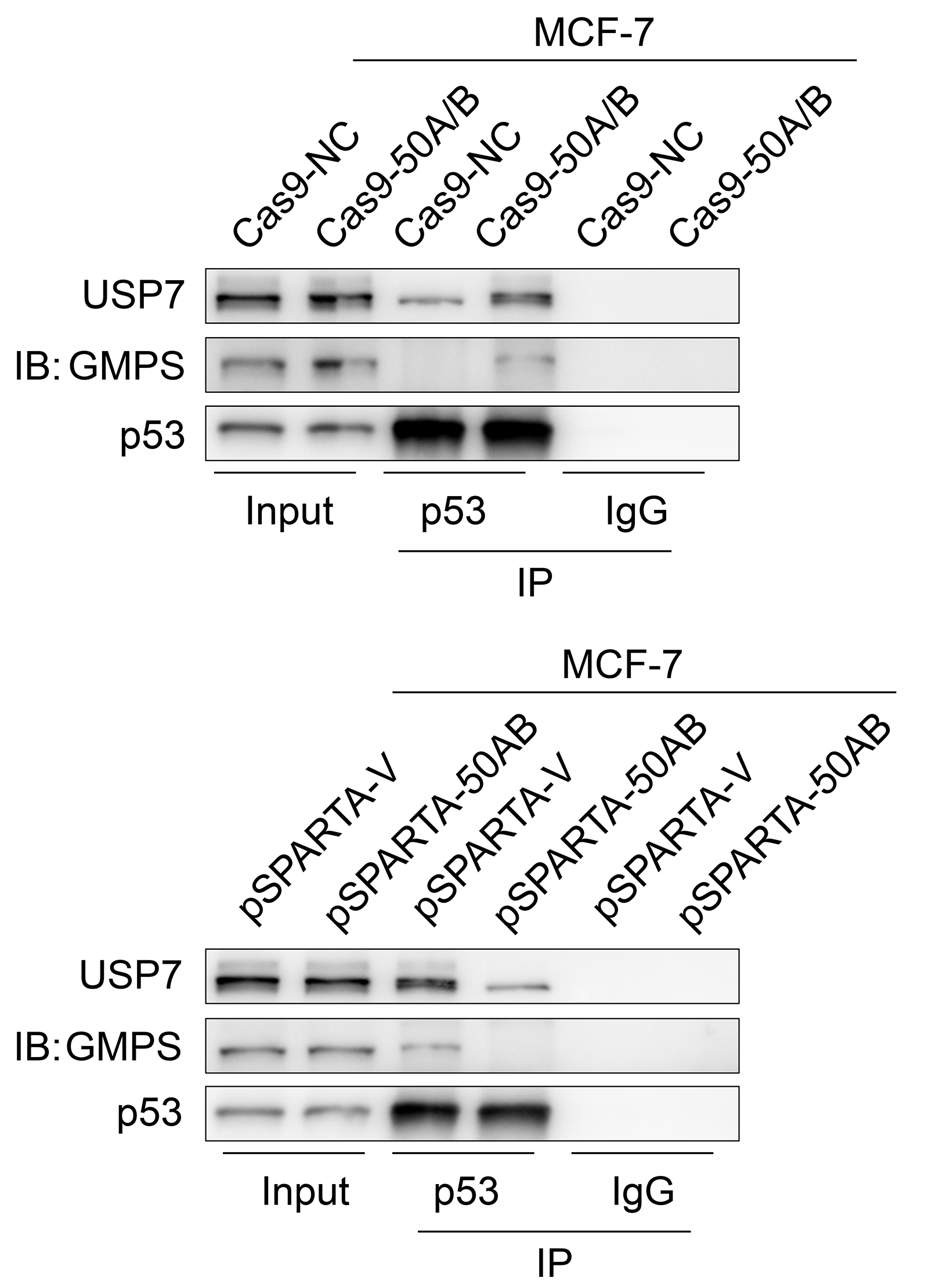

Supplement: Supplementary file 13 — Figure S12 [file 41418_2021_762_MOESM13_ESM.tif]

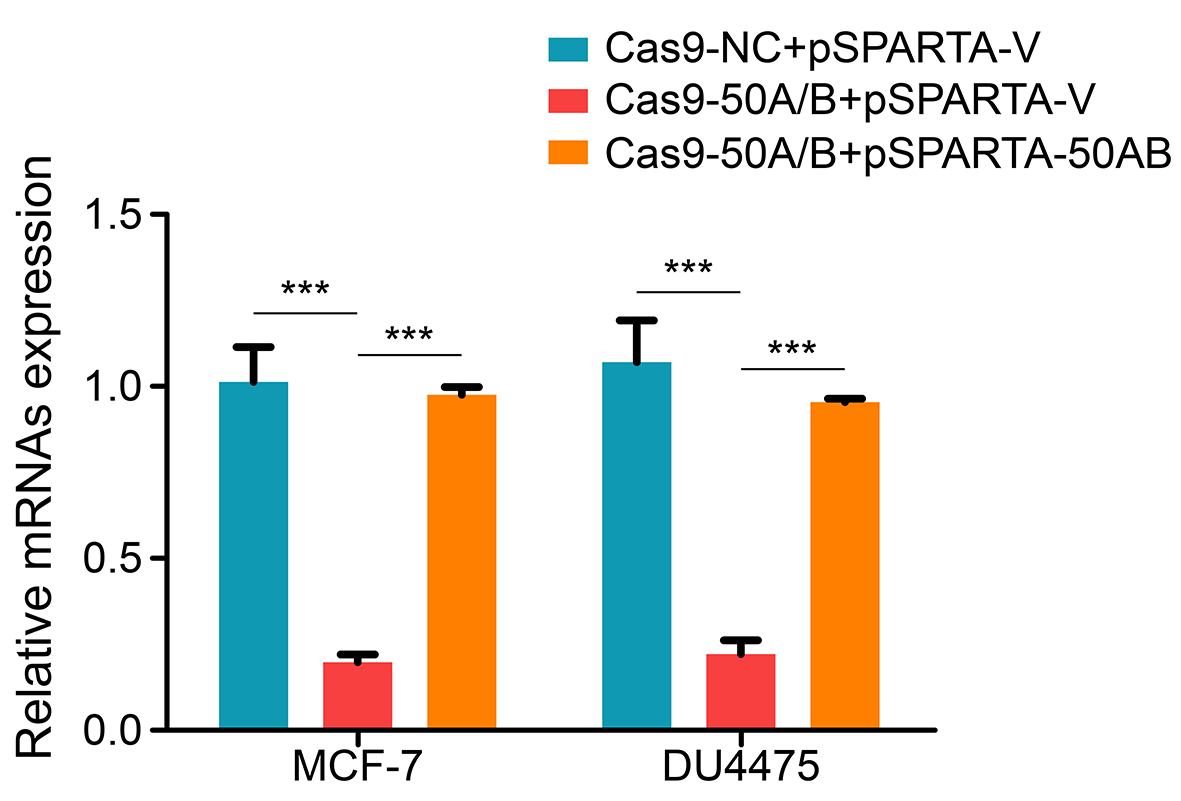

Supplement: Supplementary file 14 — Figure S13 [file 41418_2021_762_MOESM14_ESM.tif]

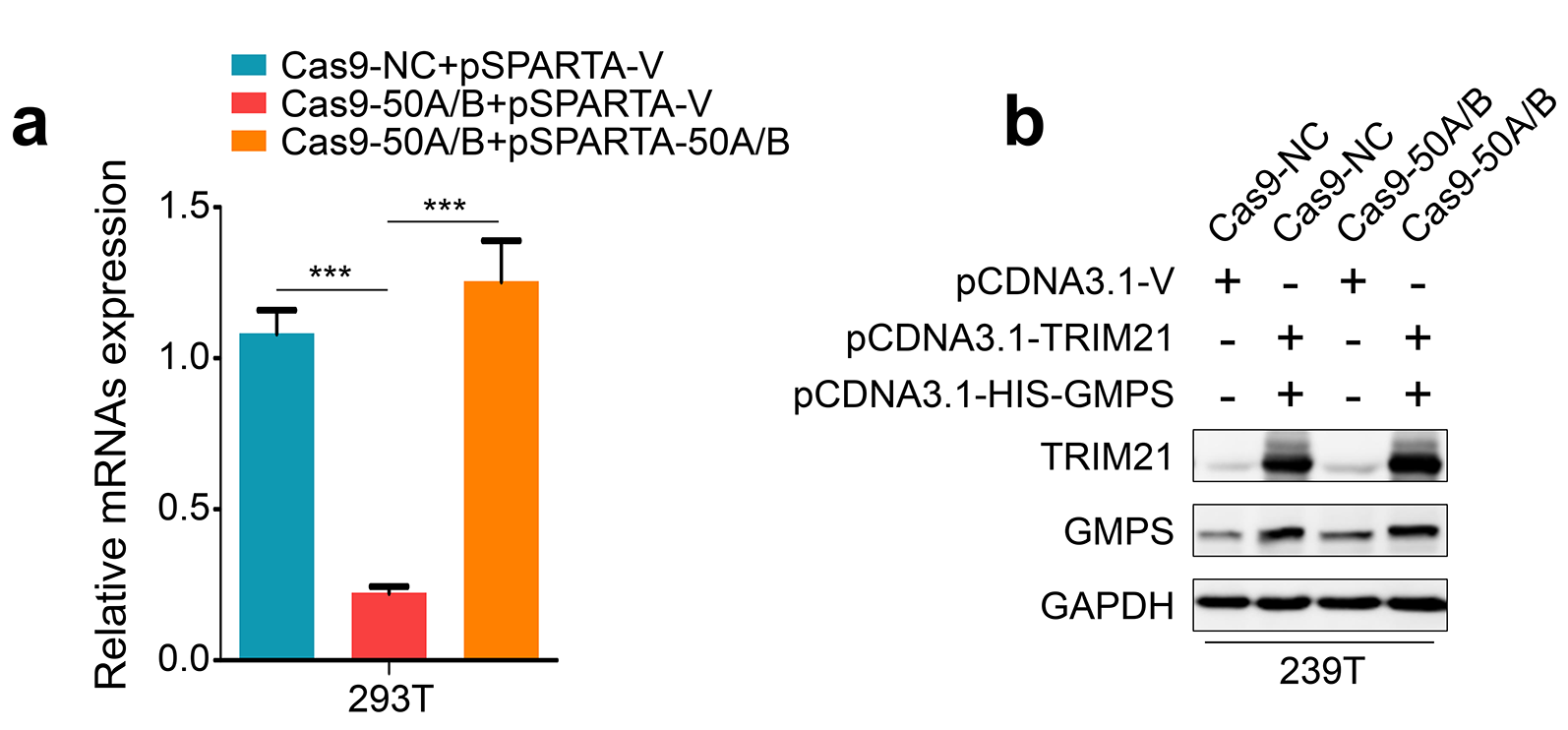

Supplement: Supplementary file 15 — Figure S14 [file 41418_2021_762_MOESM15_ESM.tif]

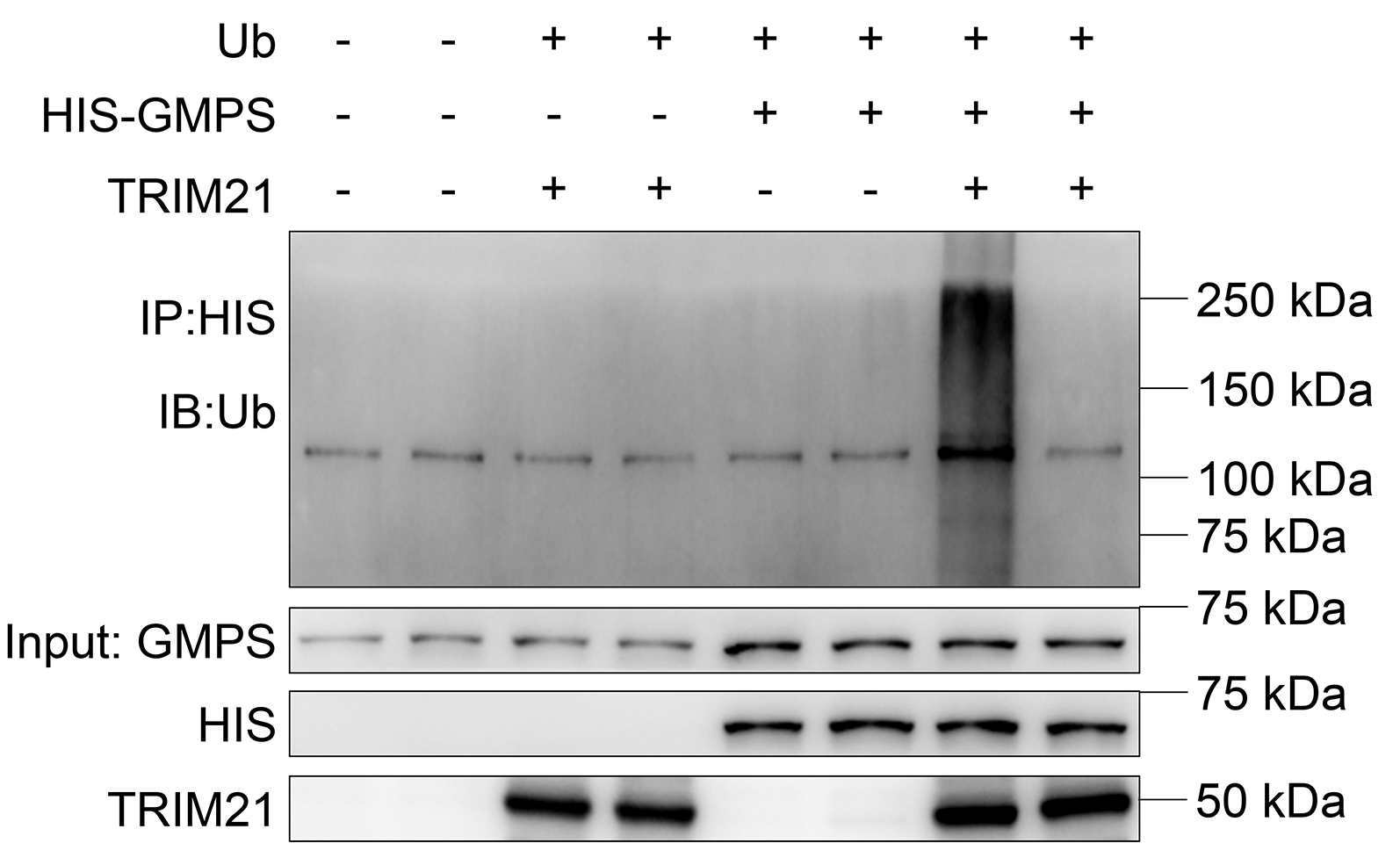

Supplement: Supplementary file 16 — Figure S15 [file 41418_2021_762_MOESM16_ESM.tif]

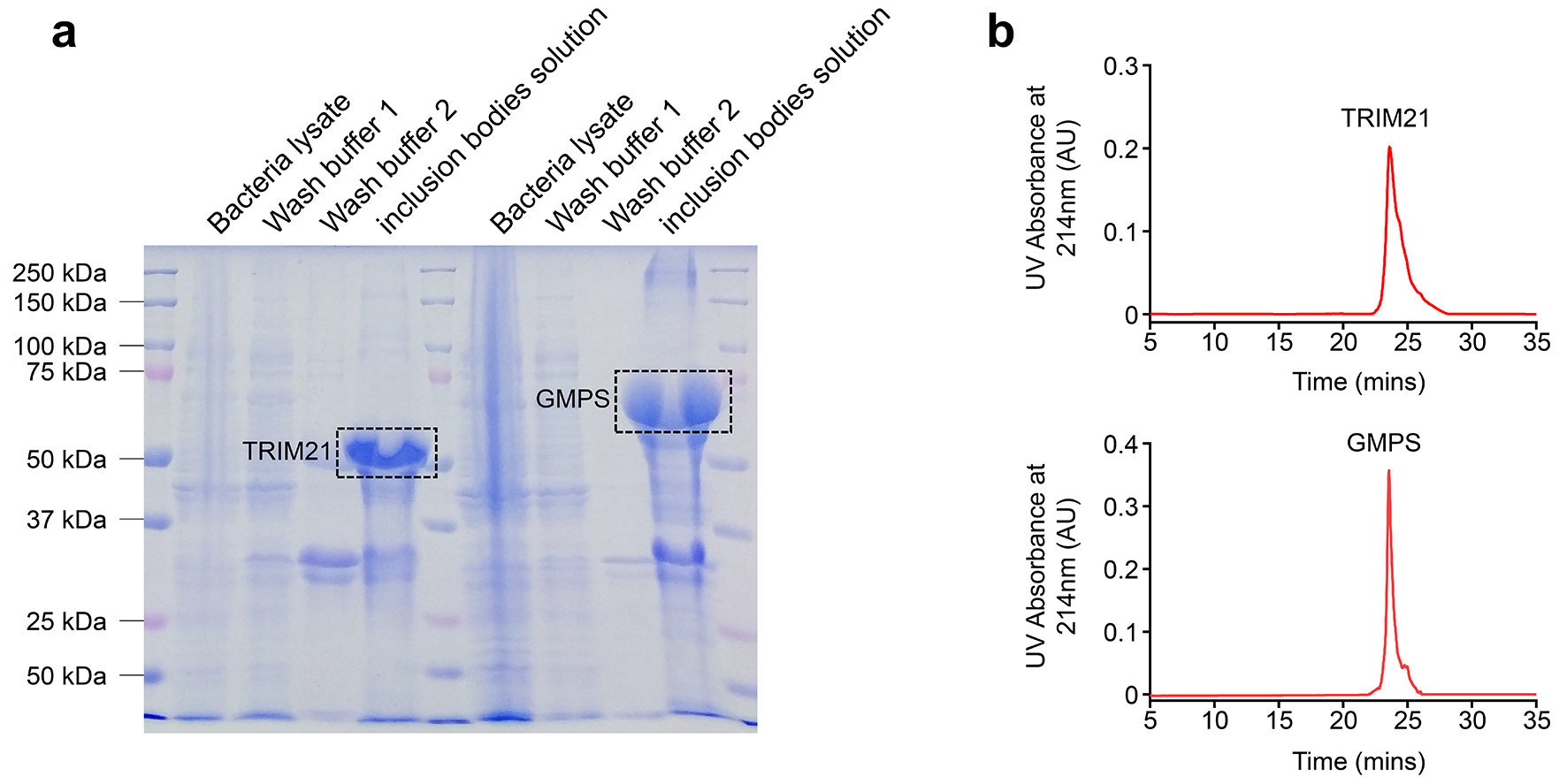

Supplement: Supplementary file 17 — Figure S16 [file 41418_2021_762_MOESM17_ESM.tif]

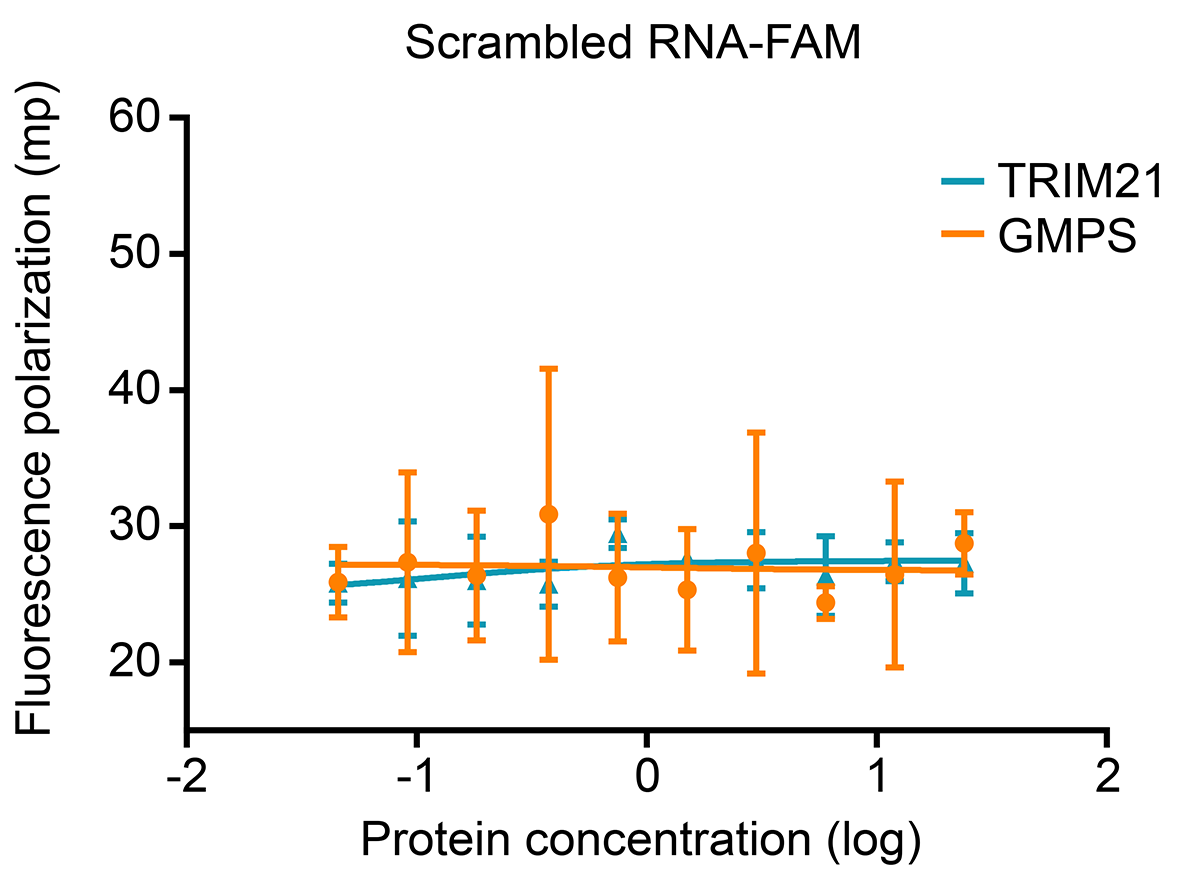

Supplement: Supplementary file 18 — Figure S17 [file 41418_2021_762_MOESM18_ESM.tif]
